# Synthesis and Antitumor Evaluation of Novel 5-Hydrosulfonyl-1*H*-benzo[*d*]imidazol-2(3*H*)-one Derivatives

**DOI:** 10.3390/molecules21040516

**Published:** 2016-04-20

**Authors:** Guang Ouyang, Rongsheng Tong, Jinqi Li, Lan Bai, Liang Ouyang, Xingmei Duan, Fengqiong Li, Pin He, Jianyou Shi, Yuxin He

**Affiliations:** 1 Individualized Medication Key Laboratory of Sichuan Province, Sichuan Academy of Medical Science & Sichuan Provincial People’s Hospital, Chinese Academy of Sciences Sichuan Translational Medicine Research Hospital, School of Medicine, Center for Information in Medicine, University of Electronic Science and Technology of China, Chengdu 610072, Sichuan, China; oyg613@163.com (G.O.); tongrs@126.com (R.T.); lijinqi2002@126.com (J.L.); blci@163.com (L.B.); duanxingmei2003@163.com (X.D.); lifengqiong2014@163.com (F.L.); hep403@163.com (P.H.); 2 Bioengineering College, Xihua University, Chengdu 610039, Sichuan, China; 3 State Key Laboratory of Biotherapy, West China Hospital, Sichuan University, Chengdu 610041, Sichuan, China; klivis@163.com

**Keywords:** antitumor activity, 5-hydrosulfonyl-1*H*-benzo[*d*]imidazol-2(3*H*)-one, synthesis

## Abstract

A series of novel 5-hydrosulfonyl-1*H*-benzo[*d*]imidazol-2(3*H*)-one derivatives bearing natural product substructures has been successfully synthesized and their antitumor activity studied. These newly synthesized derivatives were characterized by ^1^H-NMR, ^13^C-NMR and high resolution mass spectral data, then screened as antitumor agents against the A549, HCC1937, and MDA-MB-468 human tumor cell lines using MTT cell proliferation assays. The results show that some of these compounds can effectively inhibit the growth of these cancerous cells, with compound **5b** being the best one (IC_50_ = 2.6 μM). Flow cytometry data revealed that compound **5b** induced apoptosis of HCC1937 cells with increased solution concentration. The structure and activity relationships (SAR) of these compounds is summarized.

## 1. Introduction

Cancer poses a threat to worldwide health due to its high mortality. Environmental degradation has been associated with increased cancer incidence [1]. It is estimated that the global incidence of cancer in both men and women will increase from an estimated 12.7 million cases in 2008 to 20.3 million by 2030, a net increase of about 60% [2]. Therefore, it is very urgent to develop new, high effective and safe antitumor drugs.

**Figure 1:**
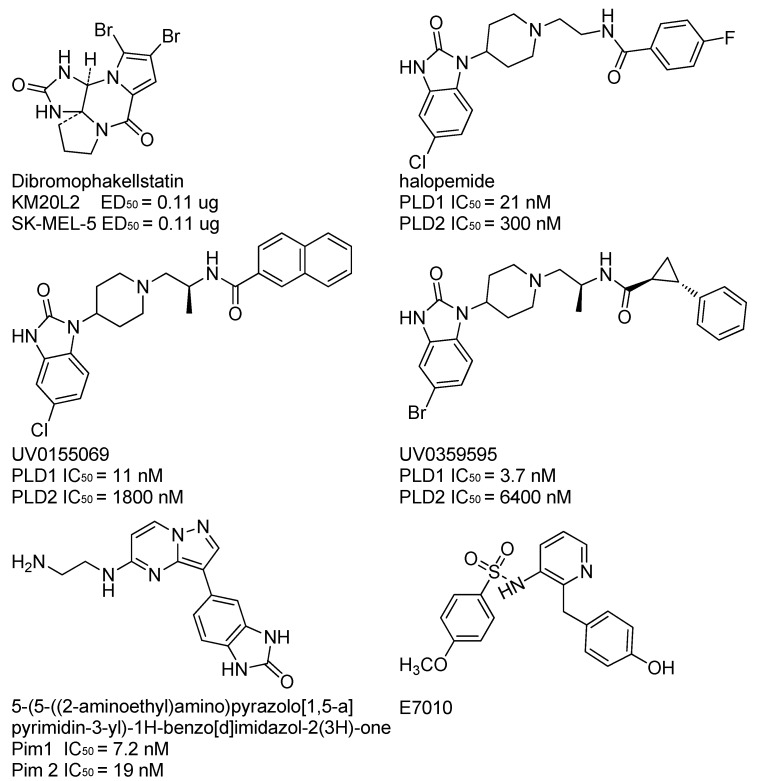
Structures and activities of dibromophakellstatin 1A, halopemide 2A, isoform-selective PLD (PLD1 selective) inhibitors 3A and 4A, Pim 1/2 inhibitor 5-(5-((2-aminoethyl)amino)pyrazolo[1,5-*a*]pyrimidin-3-yl)-1*H*-benzo[d]imidazol-2(3*H*)-one, tubulin inhibitor E7010.

Dibromophakellstatin (Figure 1), a natural product isolated from *Phakellia mauritiana*, has good antitumor activity [3]. Dibromophakellstatin, a new cancer cell growth inhibitor, has a dihydrobenzoimidazol-2-one scaffold [4]. Other dihydro-benzoimidazol-2-one analogues have been found to be effective compounds when used as hypoglycemia agents [5], muscarinic acetylcholine esterase inhibitors [6], antimicrobial agents [7] and pigments [8]. In recent years, dihydro-benzoimidazol-2-one, was used as a novel scaffold in the tumor therapeutic area, and attentions on it has been increasing. Dihydrobenzoimidazol-2-one derivatives show increased anti-tumor activity by inhibiting different receptors [9,10,11,12,13,14], now there has been great progress in the study of dihydro-benzoimidazol-2-one derivatives. Thus, halopemide [15] (Figure 1) is the first potent, direct-acting, drug-like, small-molecule phospholipase D1/2 (PLD1/2) inhibitor; UV01555069 and UV0359595 are potent selective phospholipase D1 (PLD1) inhibitors [16,17]; 5-(5-((2-aminoethyl)amino)pyrazolo[1,5-*a*]pyrimidin-3-yl)-1*H*-benzo[d]imidazol-2(3*H*)-one is a Pim-1/2 kinase inhibitor [18]. Other effects, such as reversible Cdc25 inhibition [19]; dual CCR3/H1 antagonism [20]; and non-thiolfarnesyltransferase inhibition [21] are also reported. In the above studies, structural modifications were mainly performed at the 1-, 3- and 5-positions of the dihydrobenzoimidazol-2-one ring. The sulfonamide group is an important pharmacophore that often appears in antitumor drugs. E7010 [22], for example, is a kind of tubulin inhibitor containing a sulfonamide group and it was shown that the sulfonamide group played an important role in its activity. To the best of our knowledge, however, the replacement of a sulfamide group at the 5-position of the dihydrobenzoimidazol-2-one scaffold is not reported.

Because dihydrobenzoimidazol-2-one derivatives have potential antitumor activity, we focused our attention on introducing proper functional groups into the dihydrobenzoimidazol-2-one ring. Previous studies on the dihydrobenzoimidazol-2-one scaffold showed that such compounds have potent antitumor activity, so in this work a series of 5-hydrosulfonyl-1*H*-benzo[*d*]imidazol-2(3*H*)-one derivatives has been designed and synthesized by introducing the sulfamide groups at the 5-position. The new compounds were tested for their anti-cancer activity against some different cell lines and it was found that compound **5b** (Table 1) has the best activity against the HCC1937 cell line.

## 2. Results and Discussion

### 2.1. Chemistry

**Scheme 1:**
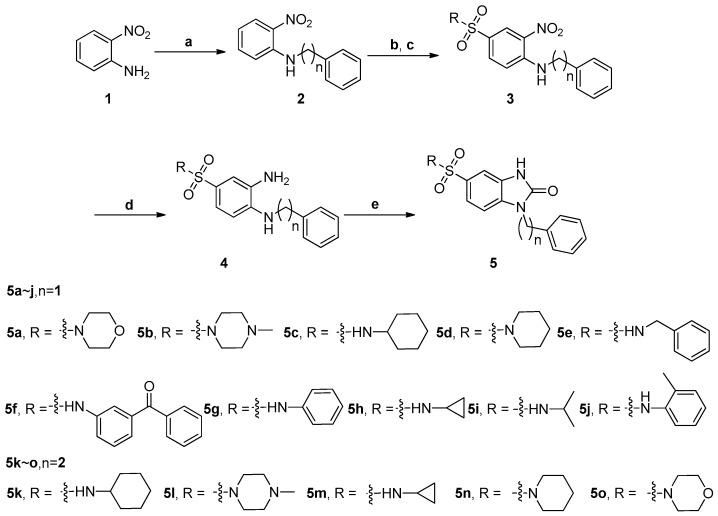
Syntheses of compounds **5a**–**o**. *Reactions and conditions*: (**a**) NaOH, benzyl bromide or (2-bromoethyl)benzene, acetone, 65 °C, 1.5 h; (**b**) chlorosulfonic acid, 0 °C, 4 h; (**c**) ammonia derivatives, THF, rt, 0.5 h; (**d**) Fe/HCl, silica gel, 80% ethyl alcohol, 80 °C, 1 h; (**e**) triphosgene, anhydrous THF, 0 °C, 0.5 h.

The novel 5-hydrosulfonyl-1*H*-benzo[*d*]imidazol-2(3*H*)-one derivatives described herein were synthesized as shown in Scheme 1. Substitution reactions of 2-nitroaniline (**1**) with benzyl bromide or (2-bromoethyl)benzene and NaOH in acetone gave compounds **2**, then chlorosulfonation with chlorosulfonic acid and another substitution reaction with ammonia derivatives in tetrahydrofuran (THF) gave compounds **3**, which were reduced by Fe/HCl to afford compounds **4**, which were then cyclized with triphosgene (BTC) in anhydrous THF to give compounds **5a**–**o**. All the newly synthesized compounds were characterized on the basis of their ^1^H-NMR, ^13^C-NMR and HRMS data.

### 2.2. Anti-Cancer Activity

Compounds **5a**–**o** were evaluated for their cytotoxic activity *in vitro* against some human cancer cell lines, including A549, HCC1937 and MDA-MB-468 by applying a MTT colorimetric assay. Doxorubicin was used as positive control. The calculated IC_50_ values were reported differently according to the different cancer cells. The results are summarized in Table 1. The data in Table 1 shows that some of the synthesized compounds exhibited distinct cytotoxic activity against the A549, HCC1937 and MDA-MB-468 human cancer cell lines *in vitro*.

### 2.3. Flow Cytometry

**Figure 2:**
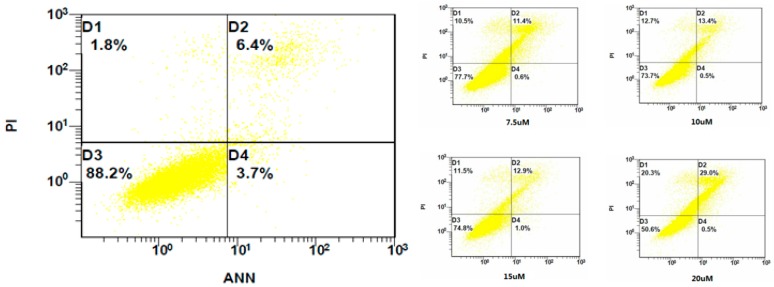
Flow cytometry results.

To exhibit a far more explicit function of compound **5b** on HCC1937 Cells, we did a cell flow experiment with HCC1937. When the concentration of **5b** was 7.5 μM, the proportion of apoptotic cells was 11.4%. When the concentration was 10 μM, the proportion of apoptotic cells was 13.4%. When the concentration was 15 μM, the proportion of apoptotic cells was 12.9%. When the concentration was 20 μM, the proportion of apoptotic cells was 29%. The proportion of apoptotic cells in control group was 6.7%. As the concentration increased, the number of apoptotic cells increased gradually and the number of apoptotic cells and concentration were directly proportional, although there was no significant difference between 10 μM and 15 μM. The results are shown in Figure 2.

**Figure 3:**
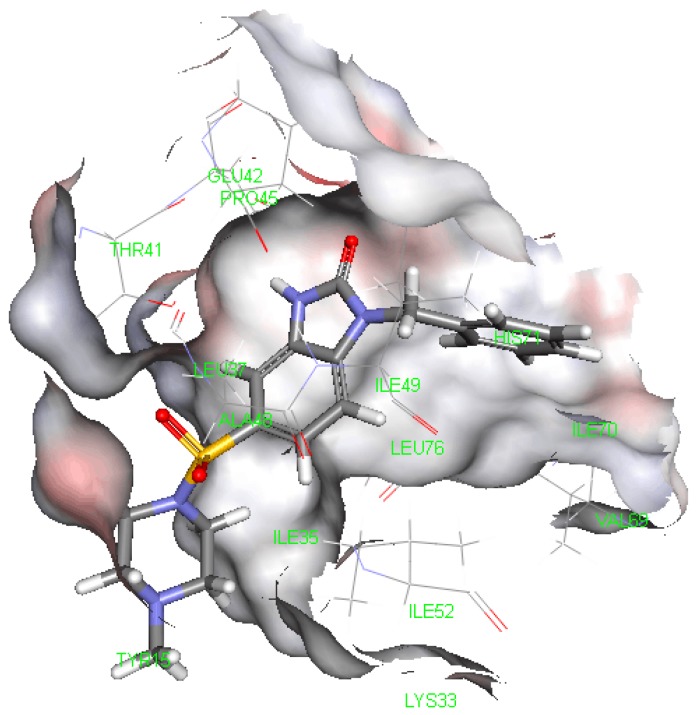
Molecular docking of **5b** and CDK2.

Using the Pharmacophores Ligand module of the Profiler DS3.1 program to reverse the drug group screening, a total of 1300 targets were screened. According to the reverse pharmacodynamic docking method, **5b** has a good effect on CDK2. The results are shown in Table 2. In the molecular docking of compound **5b** and CDK2, the oxygen atom of the sulfonic group and SER141 amino acid residues form hydrogen bond interactions, suggesting that compound **5b** has a good binding capacity to CDK2 protein. The results are shown in Figure 3.

### 2.4. Discussion

We have designed and synthesized some novel 5-hydrosulfonyl-1*H*-benzo[*d*]imidazol-2(3*H*)-one derivatives, and evaluated their antitumor activities against the A549, HCC1937 and MDA-MB-468 cell lines. Many of the new compounds displayed cytotoxicity toward the tested cell lines. The structure–activity relationship results suggest that sulfamine groups (R) and substituted phenyl or benzyl (R_1_) substituent groups in the dihydrobenzoimidazol-2-one ring are associated with the cytotoxicity activity. When R_1_ is phenyl and R is 1-methylpiperazine (compound **5a**) or tetrahydro-2*H*-pyran (compound **5b**), the activity toward the A549, HCC1937 and MDA-MB-468 cell lines is good (IC_50_ = 2.6–9 μM), whereas when R is cyclohexanamine (compound **5c**) or piperidine (compound **5d**) the resulting compound exhibits equivalent to 10-fold inferior potency compared to **5a** and **5b**. On the other hand, the aliphatic moieties **5h** and **5i** and the various amino moieties found in **5e**, **5f**, **5g** and **5j** resulted in almost no activity. When R_1_ is benzyl, various R moieties exhibited similar activity as when R_1_ was phenyl. Further studies on additional related structural modifications are currently in progress in our laboratory. To investigate the apoptotic effects in HCC1937 cells following stimulation with 7.5, 10, 15 or 20 μmol/L of **5b**, flow cytometry analyses were used. The data revealed that the **5b**-induced apoptosis rate of HCC1937 cells increased from 11.4% to 29% as the concentration increased.

## 3. Materials and Methods

### 3.1. General Information

Chemical reagents were obtained from commercial suppliers, and were dried and purified by standard methods when necessary. The progress of reactions was monitored by thin layer chromatography (TLC) using silica gel plates. The ^1^H-NMR and ^13^C-NMR spectra were recorded on a Bruker (Bruker, Rheinstetten, Germany) AVANCE III400 Hz spectrometer using CDCl_3_ or DMSO-*d*_6_ as solvent. Tetramethylsilane (δ = 0.00 ppm) was used as an internal standard. HRMS data were obtained using Bruker micrOTOF-Q instrument or TOF-MS instrument.

### 3.2. Synthesis

The preparation of the compounds **5a**–**o** was accomplished by the series of steps described below for the preparation of **5a**. 

#### 3.2.1. *N*-Benzyl-2-Nitroaniline (**2a**)

To a solution of 2-nitroaniline (**1**, 1.38 g, 10 mmol) and NaOH (480 mg, 12 mmol) in acetone (20 mL), the reaction mixture was maintained at 65 °C over 15 min while stirring, then benzyl bromide (1.43 mL, 12 mmol) was added dropwise over 5 min. After the suspension was stirred at 65 °C for 1 h, the reaction mixture was poured into water and extracted with ethyl acetate (3 × 15 mL). The combined organic layers were washed by brine (3 × 15 mL) and dried over anhydrous MgSO_4_. The solvent was removed *in vacuo*, and the residue was purified by flash chromatography on silica gel to obtain **2a** (2.1 g, 92%) as a yellow solid. ^1^H-NMR (DMSO-*d*_6_) δ ppm: 8.66 (s, 1H, NH), 8.08 (d, 1H, ArH), 7.45 (t, 1H, ArH), 7.39–7.33 (m, 4H, ArH), 7.25 (t, 1H, ArH), 6.92 (d, 1H, ArH), 6.67 (t, 1H, ArH) 4.63 (d, 2H, CH_2_). ^13^C-NMR (DMSO-*d*_6_) δ ppm: 144.89, 138.49, 136.37, 131.29, 128.55, 128.55, 127.04, 126.91, 126.91, 126.19, 115.39, 114.88, 45.68. HRMS: calcd. for C_13_H_12_N_2_O_2_^+^ [M + Na]^+^: 251.0796, found: 251.0798. Melting point: 73.8−74.9 °C. ^1^H-NMR, ^13^C-NMR and HRMS spectra are provided in the Supplementary Materials.

#### 3.2.2. *N*-Benzyl-4-(morpholinosulfonyl)-2-nitroaniline (**3a**)

Chlorosulfonic acid (10 mL) was cooled at 0 °C for 10 min, then the compound **2a** (2.28 g, 10 mmol) was added slowly. After stirring for 4 h at 0 °C, the reaction mixture was poured into ice water (50 mL), and then extracted with ethyl acetate (3 × 15 mL). The organic layer was dried over anhydrous MgSO_4_ and concentrated *in vacuo*. The residue was purified by flash chromatography on silica gel to obtain the intermediate. A solution of the intermediate (327 mg, 1 mmol) and morpholine (87 μL, 1 mmol) in tetrahydrofuran (5 mL) was stirred for 0.5 h at room temperature, then the solvent was removed *in vacuo*. The residual solid was purified by flash chromatography on silica gel to give compound **3a** (358 mg, 65%) as a yellow solid. Melting point: 80.1−81.7 °C. ^1^H-NMR (DMSO-*d*_6_) δ ppm: 8.78 (t, 1H, NH), 8.09 (d, 1H, ArH), 7.77–7.60 (m, 4H, ArH), 7.43 (t, 1H, ArH), 6.90 (d, 1H, ArH), 6.69 (t, 1H, ArH), 4.78 (d, 2H, CH_2_), 3.57 (s, 4H, CH_2_), 2.75 (s, 4H, CH_2_). ^13^C-NMR (DMSO-*d*_6_) δ ppm: 144.47, 140.61, 136.27, 134.46, 131.98, 131.98, 131.64, 131.64, 129.68, 126.27, 125.86, 114.96, 65.18, 65.18, 45.77, 45.77, 45.02. HRMS: calcd. for C_17_H_19_N_3_O_5_S^+^ [M + Na]^+^: 400.0943, found: 400.0946.

#### 3.2.3. *N*′-Benzyl-4-(morpholinosulfonyl)benzene-1,2-diamine (**4a**)

A mixture of silica gel (1.5 g, 4 equivalents), and iron dust (140 mg, 2.5 mmol) was added to an ethyl alcohol/water (4:1) mixture (10 mL), the mixture was adjusted to pH 4~5 with 1 N HCl. After stirring for 10 min at 80 °C, compound **3a** (377 mg, 1 mmol) was added. The reaction mixture was stirred for 1 h at the same temperature and filtered, the filtrate was adjusted to pH 8~9 with saturated NaHCO_3_, some silica gel was added to the mixture and filtered. The mixture was added to the water (100 mL), and then extracted with ethyl acetate (3 × 15 mL). The organic layer was dried over anhydrous MgSO_4_ and concentrated *in vacuo*. The residue was purified by flash chromatography on silica gel to obtain **4a** (277 mg, 80%) as a brown solid. Melting point: 178.3−179.4 °C. ^1^H-NMR (DMSO-*d*_6_) δ ppm: 7.73 (d, 1H, ArH), 7.65–7.54 (m, 3H, ArH), 6.55 (d, 1H, ArH), 6.35–6.34 (m, 2H, ArH), 6.26 (d, 1H, ArH), 5.35 (t, 1H, NH), 4.58 (s, 2H, NH_2_), 4.44 (d, 2H, CH_2_), 3.55 (t, 4H, CH_2_), 2.68 (s, 4H, CH_2_). ^13^C-NMR (DMSO-*d*_6_) δ ppm: 142.41, 135.46, 134.77, 134.07, 132.21, 129.25, 129.25, 126.02, 126.02, 125.70, 117.23, 114.23, 65.19, 65.19, 46.15, 45.74, 45.74. HRMS: calcd. for C_17_H_21_N_3_O_3_S^+^ [M + H]^+^: 348.1382, found:348.1368. 

#### 3.2.4. Benzyl-5-(morpholinosulfonyl)-1*H*-benzo[*d*]imidazol-2(3*H*)-one (**5a**)

To a solution of **4a** (347 mg, 1 mmol) in anhydrous tetrahydrofuran (3 mL) at 0 °C for 10 min, a solution of triphosgene (148 mg, 0.6 mmol) in anhydrous tetrahydrofuran (3 mL) was added dropwise at the same temperature. After stirring for 30 min, the reaction mixture was poured into water (30 mL) and adjusted to pH 8~9 with saturated NaHCO_3_, and then extracted with ethyl acetate (3 × 15 mL). The organic layer was dried over anhydrous MgSO_4_ and concentrated *in vacuo*. The residue was purified by flash chromatography on silica gel to afford **5a** (280 mg, 75%) as a white solid. Melting point: 198.5−200.1 °C. ^1^H-NMR (DMSO-*d*_6_) δ ppm: 11.02 (s, 1H, NH), 7.65 (d, 4H, ArH), 7.08–6.93 (m, 4H, ArH), 5.16 (s, 2H, CH_2_), 3.57 (s, 4H, CH_2_), 2.76 (s, 4H, CH_2_). ^13^C-NMR (DMSO-*d*_6_) δ ppm: 154.33, 138.89, 134.64, 132.20, 129.89, 128.29, 128.29, 126.59, 126.59, 126.24, 121.25, 120.59, 108.98, 65.17, 65.17, 45.75, 42.67, 42.67. HRMS: calcd. for C_18_H_19_N_3_O_4_S^+^ [M + Na]^+^: 396.0994, found: 396.0992. 

The following compounds were similarly prepared:

*Benzyl-5-(4-methylpiperazin-1-ylsulfonyl)-1H-benzo[d]imidazol-2(3H)-one* (**5b**). Melting point: 107.2−108.1 °C. ^1^H-NMR (DMSO-*d*_6_) δ ppm: 11.01 (s, 1H, NH), 7.64 (d, 4H, ArH), 7.08–6.93 (m, 4H, ArH), 5.15 (s, 2H, CH_2_), 2.79 (s, 4H, CH_2_), 2.27 (s, 4H, CH_2_), 2.10 (s, 3H, CH_3_). ^13^C-NMR (DMSO-*d*_6_) δ ppm: 154.32, 138.84, 135.24, 132.03, 129.78, 128.28, 128.28, 126.46, 126.46, 126.17, 121.22, 120.58, 108.95, 53.33, 53.33, 45.57, 45.57, 45.20, 42.66. HRMS: calcd. for C_19_H_22_N_4_O_3_S^+^ [M + H]^+^:387.1491, found: 387.1456. 

*Benzyl-N-cyclohexyl-2-oxo-2,3-dihydro-1H-benzo[d]imidazole-5-sulfonamide* (**5c**). Melting point: 166.7−167.2 °C. ^1^H-NMR (DMSO-*d*_6_) δ ppm: 11.01 (d, 1H, NH), 7.78–7.46 (m, 5H, ArH, NH), 6.91–6.98 (m, 4H, ArH), 5.11 (s, 2H, CH_2_), 2.82 (s, 1H, CH), 1.49–1.41 (m, 5H, CH_2_), 1.09–1.00 (m, 5H, CH_2_). ^13^C-NMR (DMSO-*d*_6_) δ ppm: 154.29, 142.62, 138.43, 131.13, 129.71, 128.31, 128.31, 126.61, 126.61, 125.28, 121.12, 120.51, 108.89, 52.04, 52.04, 42.82, 33.10, 33.10, 24.76, 24.28. HRMS: calcd. for C_20_H_23_N_3_O_3_S^+^ [M + Na]^+^: 408.1358, found: 408.1363. 

*Benzyl-5-(piperidin-1-ylsulfonyl)-1H-benzo[d]imidazol-2(3H)-one* (**5d**). Melting point: 189.4−190.5 °C. ^1^H-NMR (CDCl_3_) δ ppm: 9.86 (s, 1H, NH), 7.69 (t, 2H, ArH), 7.55–7.47 (m, 2H, ArH), 7.14–6.98 (m, 3H, ArH), 6.81 (d, 1H, ArH), 5.18 (s, 2H, CH_2_), 2.91 (t, 4H, CH_2_), 1.71 (s, 2H, CH_2_), 1.59–1.56 (m, 4H, CH_2_). ^13^C-NMR (CDCl_3_) δ ppm: 155.53, 137.55, 137.05, 131.38, 129.63, 127.96, 127.96, 127.00, 127.00, 126.30, 122.12, 121.55, 109.90, 46.87, 46.87, 44.04, 25.12, 25.12, 23.43. HRMS: calcd. for C_19_H_21_N_3_O_3_S^+^ [M + H]^+^: 372.1382, found: 372.1400. 

*N,1-Dibenzyl-2-oxo-2,3-dihydro-1H-benzo[d]imidazole-5-sulfonamide* (**5e**). Melting point: 160.6–185.9 °C. ^1^H-NMR (DMSO-*d*_6_) δ ppm: 11.00 (s, 1H, NH), 8.16 (t, 1H, ArH), 7.75 (s, 1H, ArH), 7.67 (d, 1H, ArH), 7.56–7.50 (m, 2H, ArH), 7.24–7.14 (m, 4H, ArH), 7.05–6.94 (m, 4H, ArH), 5.09 (s, 2H, CH_2_), 3.92 (d, 2H, CH_2_), 3.17 (d, 1H, NH). ^13^C-NMR (DMSO-*d*_6_) δ ppm: 154.30, 141.11, 138.47, 137.42, 131.16, 129.80, 128.32, 128.32, 128.14, 128.14, 127.50, 127.50, 127.07, 127.07, 125.46, 125.46, 125.17, 121.18, 120.60, 46.09, 42.81. HRMS: calcd. for C_21_H_19_N_3_O_3_S^+^ [M + Na]^+^: 416.1045, found: 416.1030. 

*N-(3-Benzoylphenyl)-1-benzyl-2-oxo-2,3-dihydro-1H-benzo[d]imidazole-5-sulfonamide* (**5f**). Melting point: 85.2−86.3 °C. ^1^H-NMR (DMSO-*d*_6_) δ ppm: 11.00 (d, 1H, NH), 10.00 (s, 1H, NH), 7.64–6.89 (m, 17H, ArH), 5.00 (d, 2H, CH_2_). ^13^C-NMR (CDCl_3_) δ ppm: 198.58, 155.08, 139.47, 138.78, 137.64,137.56, 133.84, 133.21, 132.80, 131.87, 129.78, 128.26, 128.26, 128.21, 127.83, 127.66, 126.60, 126.60, 126.18, 126.18, 125.96, 123.54, 122.70, 122.07, 121.79, 109.64, 43.79. HRMS: calcd. for C_27_H_21_N_3_O_4_S^+^ [M + Na]^+^: 506.1150, found: 506.1171. 

*Benzyl-2-oxo-N-phenyl-2,3-dihydro-1H-benzo[d]imidazole-5-sulfonamide* (**5g**). Melting point: 91.9−93.3 °C. ^1^H-NMR (DMSO-*d*_6_) δ ppm: 10.98 (s, 1H, NH), 10.27 (s, 1H, NH), 7.72–7.40 (m, 4H, ArH), 7.22–6.88 (m, 9H, ArH), 5.04 (s, 2H, CH_2_). ^13^C-NMR (DMSO-*d*_6_) δ ppm: 154.23, 139.86, 138.60, 137.39, 131.83, 129.69, 129.69, 129.58, 128.99, 128.99, 125.66, 125.66, 125.38, 124.08, 121.14, 120.17, 119.95, 119.95, 108.89, 54.87. HRMS: calcd. for C_20_H_17_N_3_O_3_S^+^ [M + Na]^+^: 402.0888, found: 402.0893. 

*Benzyl-N-cyclopropyl-2-oxo-2,3-dihydro-1H-benzo[d]imidazole-5-sulfonamide* (**5h**). Melting point: 168.6−170.3 °C. ^1^H-NMR (DMSO-*d*_6_) δ ppm: 11.03 (s, 1H, NH), 7.90 (s, 1H, NH), 7.71 (s, 2H, ArH), 7.61–7.57 (m, 2H, ArH), 7.03–6.92 (m, 4H, ArH), 5.13 (s, 2H, CH_2_), 1.99 (s, 1H, CH), 0.36–0.25 (m, 4H, CH_2_). ^13^C-NMR (DMSO-*d*_6_) δ ppm: 154.29, 140.58, 138.44, 131.40, 129.73, 129.55, 128.29, 125.66, 125.31, 121.18, 120.55, 108.92, 107.99, 42.77, 24.04, 4.99, 4.99. HRMS: calcd. for C_17_H_17_N_3_O_3_S^+^ [M + Na]^+^: 366.0888, found: 366.0860.

*Benzyl-N-isopropyl-2-oxo-2,3-dihydro-1H-benzo[d]imidazole-5-sulfonamide* (**5i**). Melting point: 173.4−174.7 °C. ^1^H-NMR (DMSO-*d*_6_) δ ppm: 11.02 (s, 1H, NH), 7.75–7.54 (m, 5H, ArH), 7.00–6.92 (m, 4H, ArH), 5.12 (s, 2H, CH_2_), 3.20–3.14 (m, 1H, NH), 0.87 (d, 6H, CH_3_). ^13^C-NMR (DMSO-*d*_6_) δ ppm: 154.30, 142.22, 138.47, 131.08, 129.73, 129.50, 129.50, 128.31, 128.31, 125.37, 124.95, 121.14, 120.54, 45.20, 41.28, 23.05, 23.05. HRMS: calcd. for C_17_H_19_N_3_O_3_S^+^ [M + Na]^+^: 368.1045, found: 368.1051.

*Benzyl-2-oxo-N-o-tolyl-2,3-dihydro-1H-benzo[d]imidazole-5-sulfonamide* (**5j**). Melting point: 223.6−225.7 °C. ^1^H-NMR (DMSO-*d*_6_) δ ppm: 10.97 (s, 1H, NH), 9.55 (d, 1H, NH), 7.62–7.42 (m, 4H, ArH), 7.09–6.85 (m, 8H, ArH), 5.04 (s, 2H, CH_2_), 1.83 (s, 3H, CH_3_). ^13^C-NMR (DMSO-*d*_6_) δ ppm: 154.22, 140.94, 138.60, 134.47, 134.08, 131.73, 130.55, 129.71, 129.46, 128.31, 128.31, 126.41, 126.41, 126.15, 125.63, 125.15, 121.14, 120.55, 108.89, 42.75, 17.38. HRMS: calcd. for C_21_H_19_N_3_O_3_S^+^ [M + Na]^+^: 416.1045, found: 416.1067. 

*N-Cyclohexyl-2-oxo-1-phenethyl-2,3-dihydro-1H-benzo[d]imidazole-5-sulfonamide* (**5k**). Melting point: 187.0−188.5 °C. ^1^H-NMR (DMSO-*d*_6_) δ 10.80 (s, 1H, NH), 7.68 (d, 2H, ArH), 7.56 (d, 1H, ArH), 7.43 (t, 2H, ArH), 7.06 (s, 1H, ArH), 6.95 (s, 3H, ArH), 4.03 (dd, 2H, CH_2_), 3.04 (s, 2H, CH_2_), 2.87 (s, 1H, CH), 1.99 (s, 1H, NH), 1.56–1.39 (m, 5H, CH_2_), 1.23–1.08 (m, 5H, CH_2_). ^13^C-NMR (DMSO-*d*_6_) δ ppm: 153.99, 142.98, 140.29, 129.98, 129.98, 129.46, 129.46, 128.08, 126.25, 120.67, 120.33, 108.62, 107.72, 59.72, 51.94, 33.52, 33.13, 33.13, 26.30, 24.83, 24.83. HRMS: calcd. for C_21_H_19_N_3_O_3_S^+^ [M + Na]^+^: 422.1514, found: 422.1471. 

*5-(4-Methylpiperazin-1-ylsulfonyl)-1-phenethyl-1H-benzo[d]imidazol-2(3H)-one* (**5l**). Melting point: 218.2−219.6 °C. ^1^H-NMR (DMSO-*d*_6_) δ ppm: 10.81 (s, 1H, NH), 7.57 (d, 2H, ArH), 7.46 (d, 2H, ArH), 6.93 (s, 4H, ArH), 4.06 (t, 2H, CH_2_), 3.07 (t, 2H, CH_2_), 2.80 (s, 4H, CH_2_), 2.34 (s, 4H, CH_2_), 2.15 (s, 3H, CH_3_). ^13^C-NMR (DMSO-*d*_6_) δ ppm: 153.99, 144.44, 132.62, 130.02, 129.80, 127.97, 127.97, 127.56, 127.56, 120.65, 120.49, 108.63, 107.62, 53.45, 53.45, 45.61, 45.61, 45.26, 40.95, 33.72. HRMS: calcd. for C_20_H_24_N_4_O_3_S^+^ [M + Na]^+^: 423.1467, found: 423.1453. 

*N-Cyclopropyl-2-oxo-1-phenethyl-2,3-dihydro-1H-benzo[d]imidazole-5-sulfonamide* (**5m**). Melting point: 217.8−218.9 °C. ^1^H-NMR (DMSO-*d*_6_) δ ppm: 10.80 (s, 1H, NH), 7.82 (s, 1H, NH), 7.67 (d, 2H, ArH), 7.44 (d, 2H, ArH), 7.03 (s, 1H, ArH), 6.93 (s, 3H, ArH), 4.07 (t, 2H, CH_2_), 3.06 (t, 2H, CH_2_), 1.96 (s, 1H, CH), 0.44–0.34 (m, 4H, CH_2_). ^13^C-NMR (DMSO-*d*_6_) δ ppm: 154.00, 143.45, 138.19, 130.00, 129.53, 128.05, 128.05, 126.81, 126.81, 120.64, 120.31, 108.61, 107.70, 40.90, 33.55, 23.97, 5.02, 5.02. HRMS: calcd. for C_20_H_24_N_4_O_3_S^+^ [M + Na]^+^: 380.1045, found: 380.1005. 

*Phenethyl-5-(piperidin-1-ylsulfonyl)-1H-benzo[d]imidazol-2(3H)-one* (**5n**). Melting point: 183.3−184.3 °C. ^1^H-NMR (DMSO-*d*_6_) δ ppm: 10.81 (s, 1H, NH), 7.56 (d, 2H, ArH), 7.44 (d, 2H, ArH), 6.97–6.90 (m, 4H, ArH), 3.07 (t, 2H, CH_2_), 2.75 (t, 4H, CH_2_), 1.99 (s, 1H, CH_2_), 1.51 (d, 4H, CH_2_), 1.37 (t, 2H, CH_2_), 1.18 (t, 1H, CH_2_). ^13^C-NMR (DMSO-*d*_6_) δ ppm: 157.26, 141.15, 138.64, 134.21, 129.19, 129.19, 129.06, 129.06, 126.36, 123.51, 122.25, 113.41, 108.10, 48.21, 48.21, 45.89, 35.33, 24.74, 24.74, 23.42. HRMS: calcd. for C_20_H_23_N_3_O_3_S^+^ [M + H]^+^: 386.1538, found: 386.1543. 

*5-(Morpholinosulfonyl)-1-phenethyl-1H-benzo[d]imidazol-2(3H)-one* (**5o**). Melting point: 102.3−104.1 °C. ^1^H-NMR (DMSO-*d*_6_) δ ppm: 10.79 (d, 1H, NH), 7.5–7.45 (m, 4H, ArH), 6.96 (dd, 4H, ArH), 4.10–4.06 (m, 2H, CH_2_), 3.60 (s, 4H, CH_2_), 3.14–3.06 (m, 2H, CH_2_), 2.71 (d, 4H, CH_2_). ^13^C-NMR (DMSO-*d*_6_) δ ppm: 154.03, 144.66, 140.23, 134.27, 134.03, 132.16, 129.32, 127.62, 125.64, 120.69, 120.31, 108.64, 107.73, 65.21, 65.21, 45.75, 45.75, 40.96, 33.72. HRMS: calcd. for C_19_H_21_N_3_O_4_S^+^ [M + Na]^+^: 410.1150, found: 410.1155. 

### 3.3. Cell Proliferation Assay

All target compounds were evaluated for their cytotoxic activities (%) *in vitro* against human cancer cell lines, including A549, HCC1937 and MDA-MB-468, by applying the MTT colorimetric assay. The cell concentration was adjusted to 2 × 10^4^/mL using complete culture medium, then 100 μL cell suspension was inoculated in each well of a 96-well culture plate and cultured for 24 h. All the target compounds were adjusted to different concentrations ranging from 1.25 μmol/L to 40 μmol/L, then 100 μL solution was added to each well of 96-well culture plate and 100 μL complete culture medium was added to the control group. Cells were placed under 5% CO_2_ at 37 °C and cultured for 48 h. Then 5 mg/mL MTT 20 μL was added to each well of 96-well culture plate and cultured for another 2~4 h. The medium was removed and 150 μL DMSO was added to each well of 96-well culture plate, then oscillated and mixed 15 min. The absorbance (A) of each well was measured in terms of optical density at a wavelength of 570 nm, each cell viability assay was performed in triplicate and taken the average value, then calculated the IC_50_. Doxorubicin was the positive control drug.

### 3.4. Flow Cytometry

The Annexin V-FITC apoptosis detection kit was used to detect apoptotic cells according to the manufacturer’s protocol. Briefly, cells were gently washed with PBS and collected using trypsinization, disaggregated to a single cell suspension and incubated with 5 μL of Annexin V-FITC and 10 μL of a PI solution for 15 min in the dark. The apoptotic cells were detected using a flow cytometer (BD, San Diego, CA, USA), then quantified and the percentage of apoptotic cells measured.

## 4. Conclusions

In conclusion, we have successfully developed an efficient method for the synthesis of a series of novel 5-hydrosulfonyl-1*H*-benzo[*d*]imidazol-2(3*H*)-one derivatives via a simple four step route. This method has the advantages of convenient operation, the ready availability of starting reagents, mild reaction conditions employed, as well as good yields. Next the prepared compounds were screened against A549, HCC1937 and MDA-MB-468 human tumor cell lines using an MTT cell proliferation assay. The results suggested that some of these compounds were effective in inhibiting these cancer cells’ growth, especially compound **5b** which was the most potent one (IC_50_ = 2.6 μM) against HCC1937. The SAR data was summarized. Unfortunately only derivatives where R_1_ was a benzyl or phenyl group could be prepared and other aromatic and heterocyclic aromatics ccould not be synthesized by us. The flow cytometry data revealed that the **5b** induced apoptosis of HCC1937 cells as the concentration increased.

## Figures and Tables

**Table 1:** *In vitro* cytotoxic activity of target compounds: **5a**–**o**. 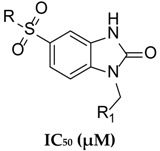

No.	Compound	MDA-MB-48	A549	HCC1937	Hek293
1	**5a**	9 ± 0.67	5.2 ± 0.35	7.8 ± 0.49	>10,000
2	**5b**	6.3 ± 0.45	3.1 ± 0.17	2.6 ± 0.21	292.6
3	**5c**	>200	88 ± 6.1	105 ± 8.9	248.2
4	**5d**	73 ± 6.9	77 ± 6.8	85 ± 7.1	756.4
5	**5e**	>200	>200	127 ± 9.9	>10,000
6	**5f**	82 ± 6.1	>200	>200	>10,000
7	**5g**	>200	>200	152 ± 9.8	2000.9
8	**5h**	>200	>200	>200	369.7
9	**5i**	>200	>200	>200	708.9
10	**5j**	>200	>200	>200	665.9
11	**5k**	135 ± 9.7	116 ± 7.6	>200	383.6
12	**5l**	22 ± 1.6	15 ± 1.1	18 ± 1.2	6312.3
13	**5m**	>200	>200	>200	447.6
14	**5n**	12 ± 1.02	16 ± 0.9	22 ± 1.3	>10,000
15	**5o**	15 ± 0.93	9.7 ± 0.88	11 ± 0.71	>10,000
**Doxorubicin**		2.7 ± 0.19	2.5 ± 0.13	4.8 ± 0.22	12.2

**Table 2:** Reverse pharmacodynamic docking results.

Pharmacophores that Fit Each Molecule						
Name	Pharmacophore	Fit Value	Class	Subclass	Family	Acronym
**5b**	1e9h-inr-2.50-h-1	0.036708	Enzymes	EC2. -(transferases)	Kinases (serine threonine)	CDK2

## Supplementary Materials

Supplementary materials can be accessed at: http://www.mdpi.com/1420-3049/21/4/516/s1. Characterization data on compounds **2a**, **3a**, **4a**, **5a**–**o**.
