# Supplementary material for: Synthesis and Antitumor Evaluation of Novel 5-Hydrosulfonyl-1H-benzo[d]imidazol-2(3H)-one Derivatives
Source: Molecules. 2016 Apr 20;21(4):516. doi: 10.3390/molecules21040516 (PMC6273811; doi:10.3390/molecules21040516)
Supplement: Supplementary file 1 [file molecules-21-00516-s001.pdf]

Supplementary Materials: Synthesis and Antitumor Evaluation of Novel 5-Hydrosulfonyl-1*H*-benzo[d]imidazol-2(3*H*)-One Derivatives

Guang Ouyang, Rongsheng Tong, Jinqi Li, Lan Bai, Liang Ouyang, Xingmei Duan, Fengqiong Li, Pin He, Jianyou Shi and Yuxin He

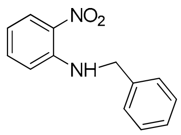

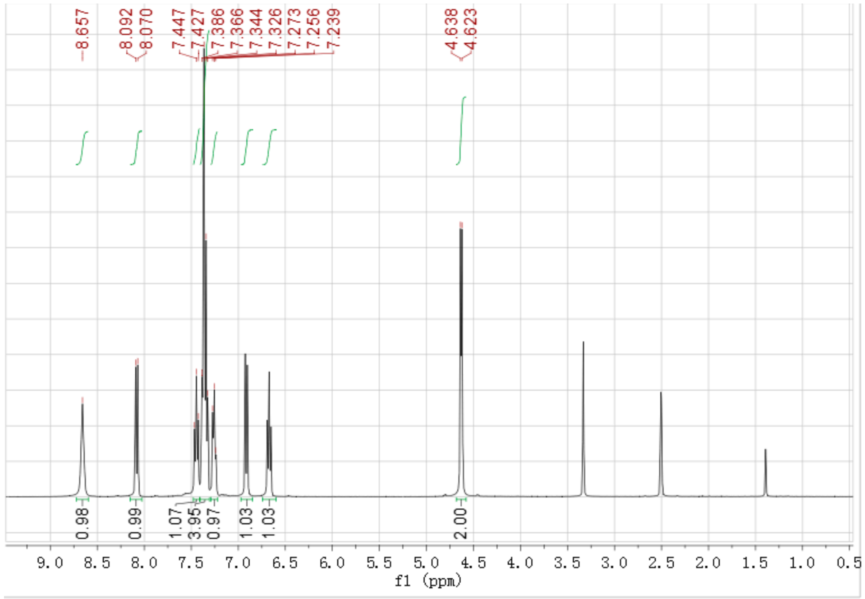

Figure S1. ^{1}H-NMR spectrum of **2a**.

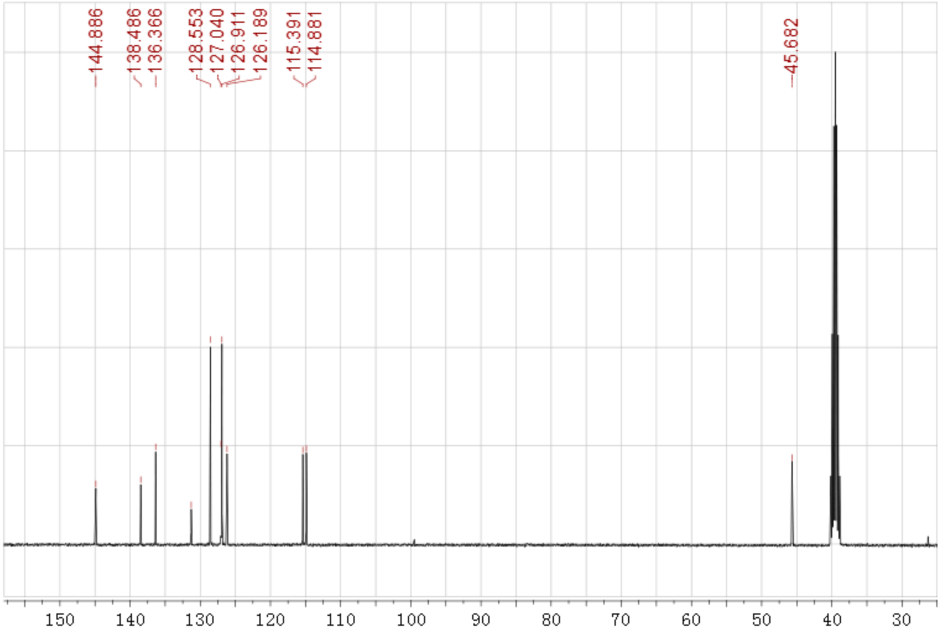

Figure S2. ^{13}C-NMR spectrum of **2a**.

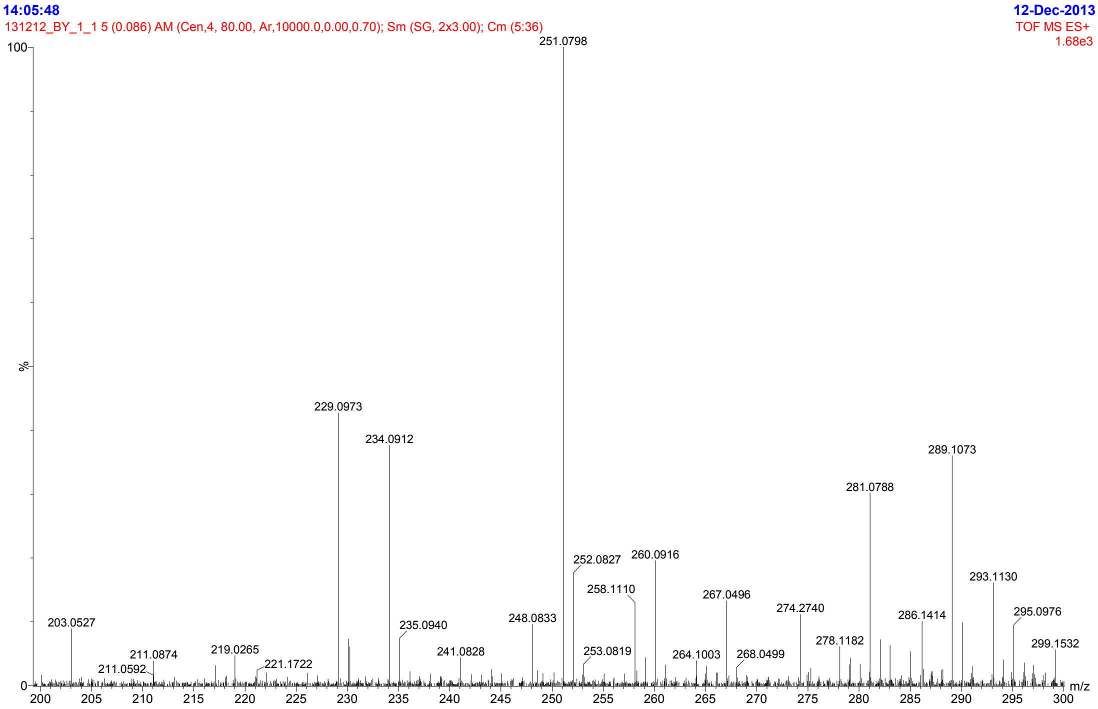

Figure S3. HRMS (EI) spectrum of **2a**.

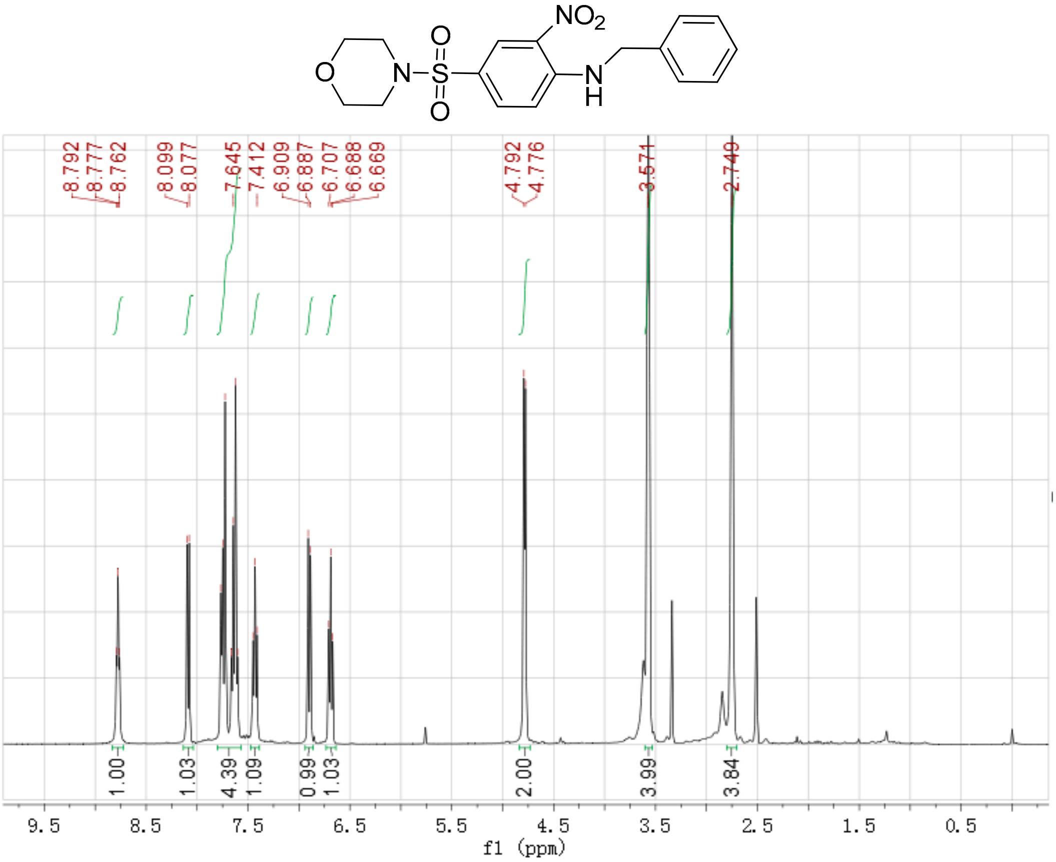

Figure S4. ^{1}H-NMR spectrum of **3a**.

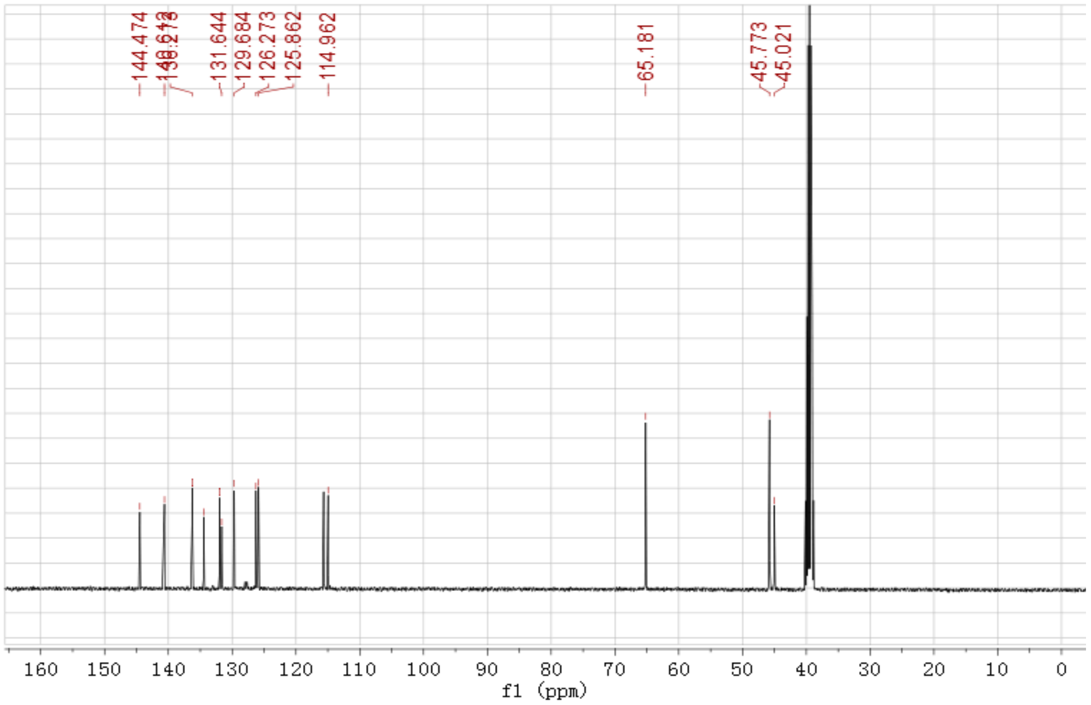

Figure S5. ^{13}C-NMR spectrum of **3a**.

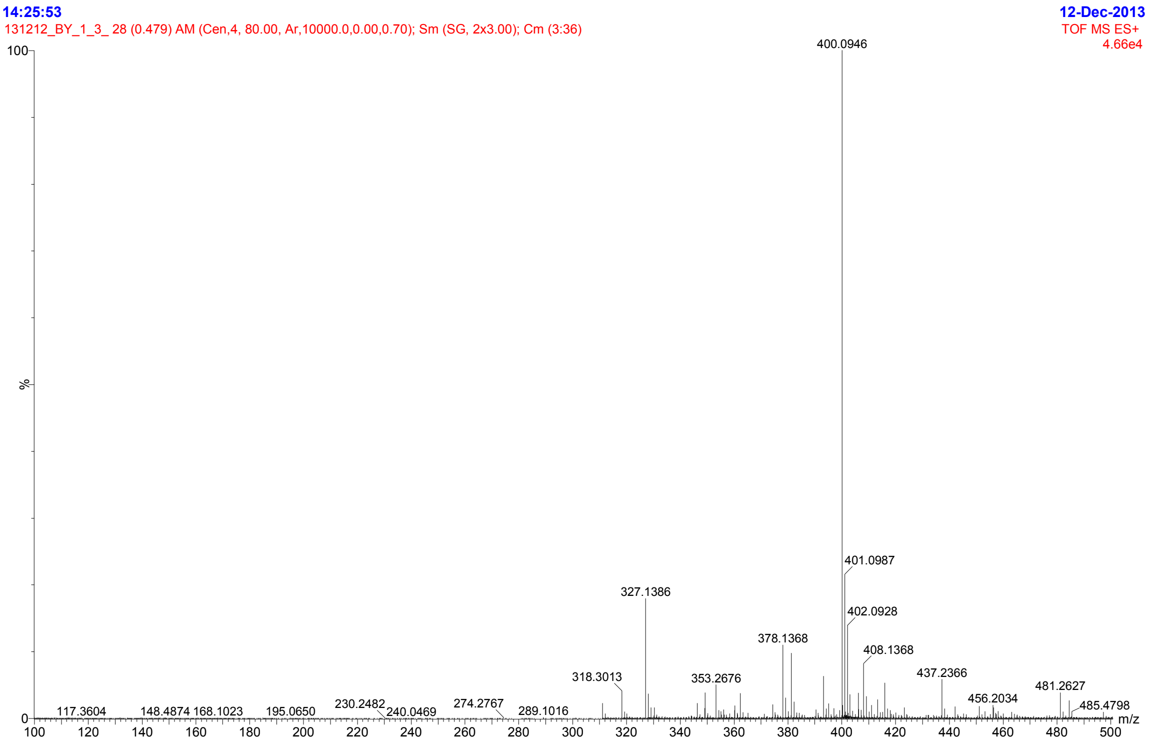

Figure S6. HRMS (EI) spectrum of **3a**.

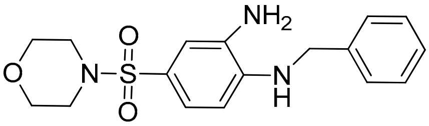

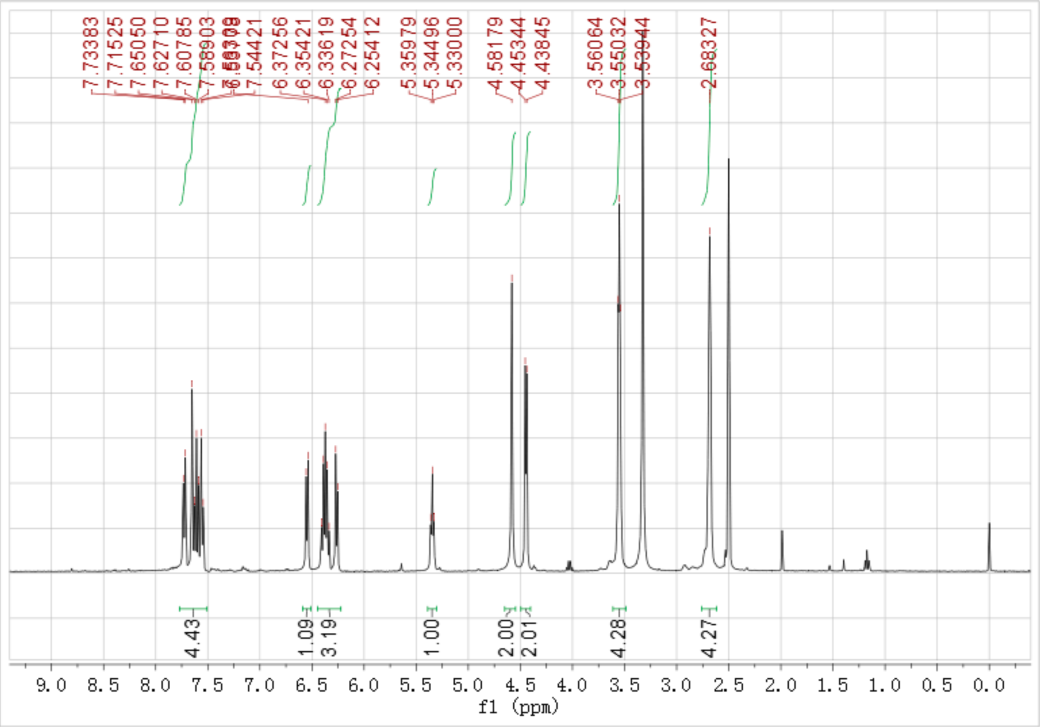

Figure S7. ^{1}H-NMR spectrum of **4a**.

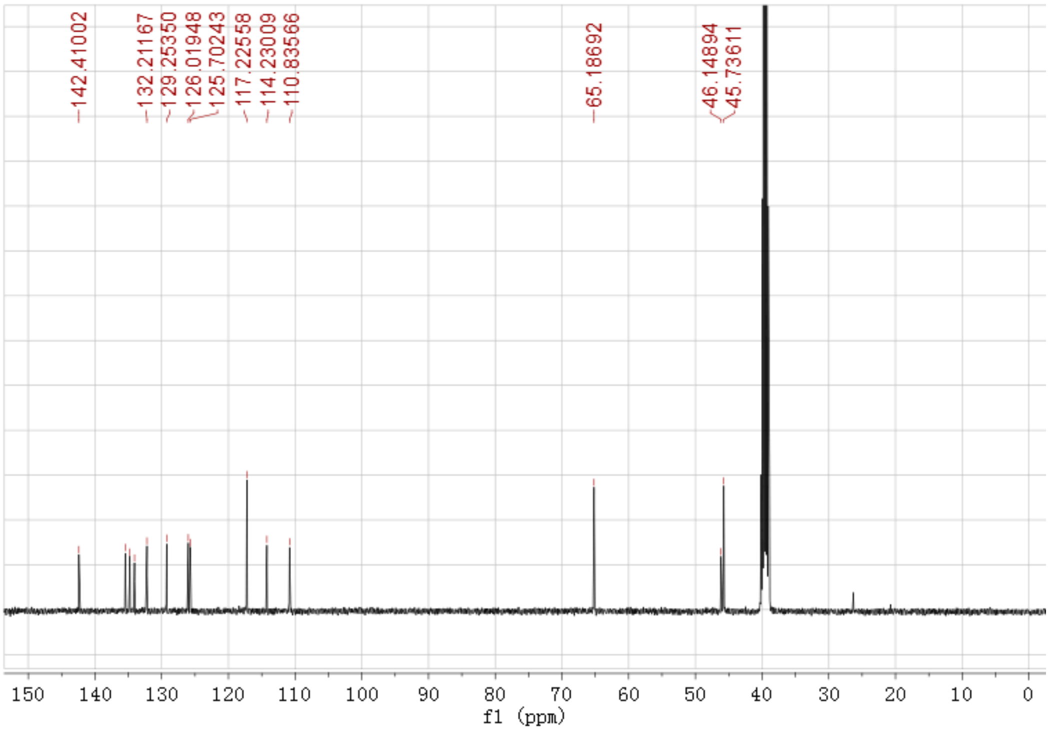

Figure S8. ^{13}C-NMR spectrum of **4a**.

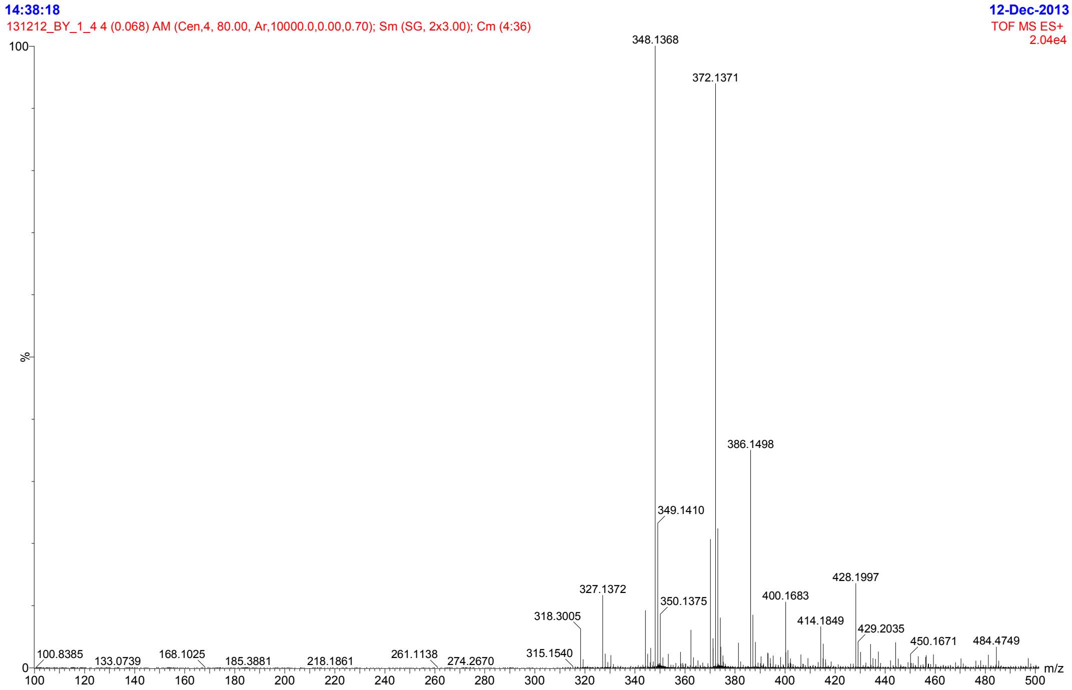

Figure S9. HRMS (EI) spectrum of **4a**.

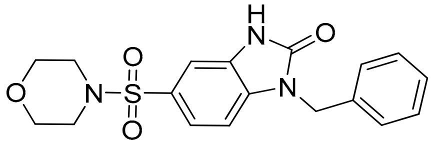

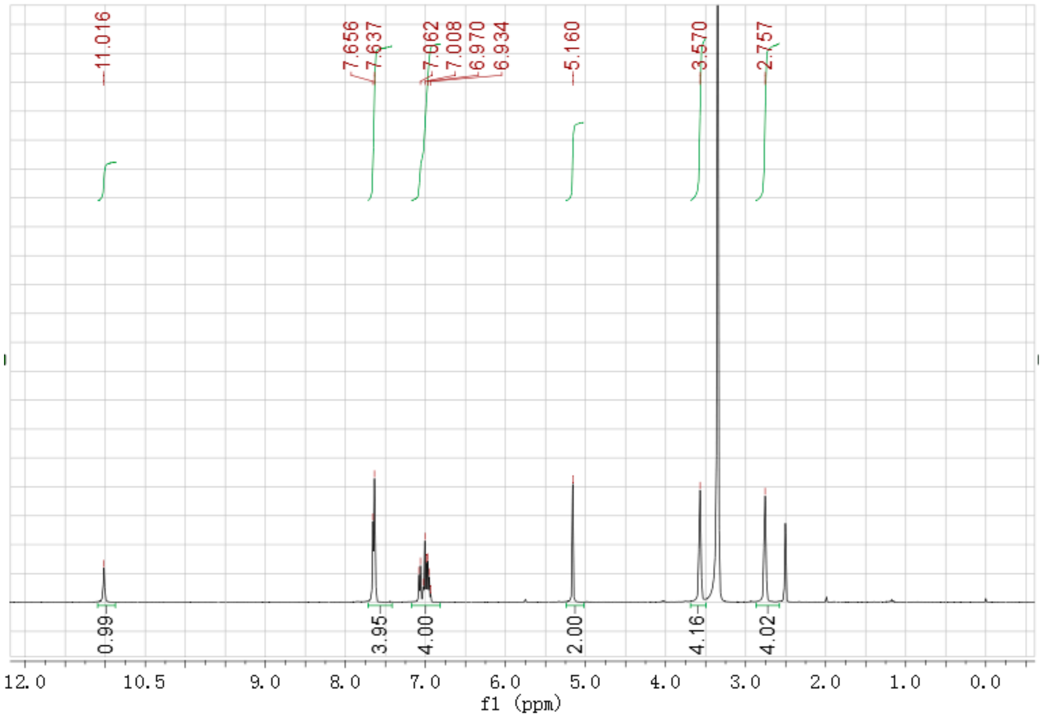

Figure S10. ^{1}H-NMR spectrum of **5a**.

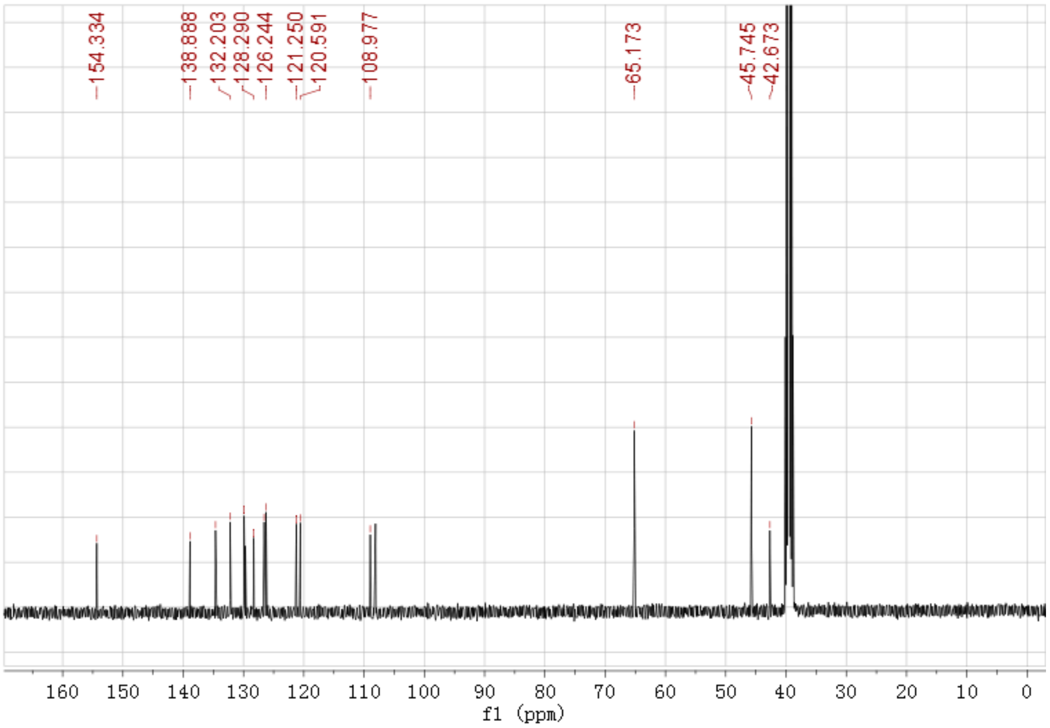

Figure S11. ^{13}C-NMR spectrum of **5a**.

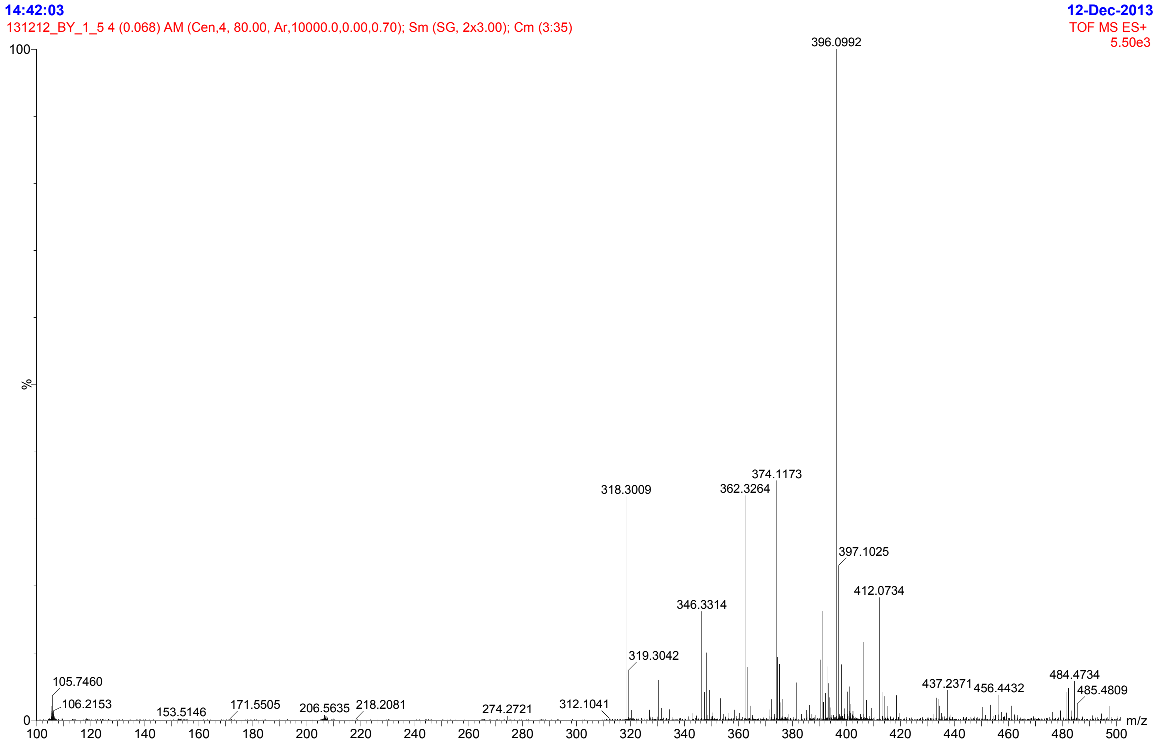

Figure S12. HRMS (EI) spectrum of **5a**.

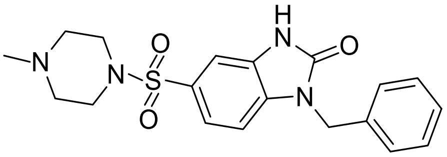

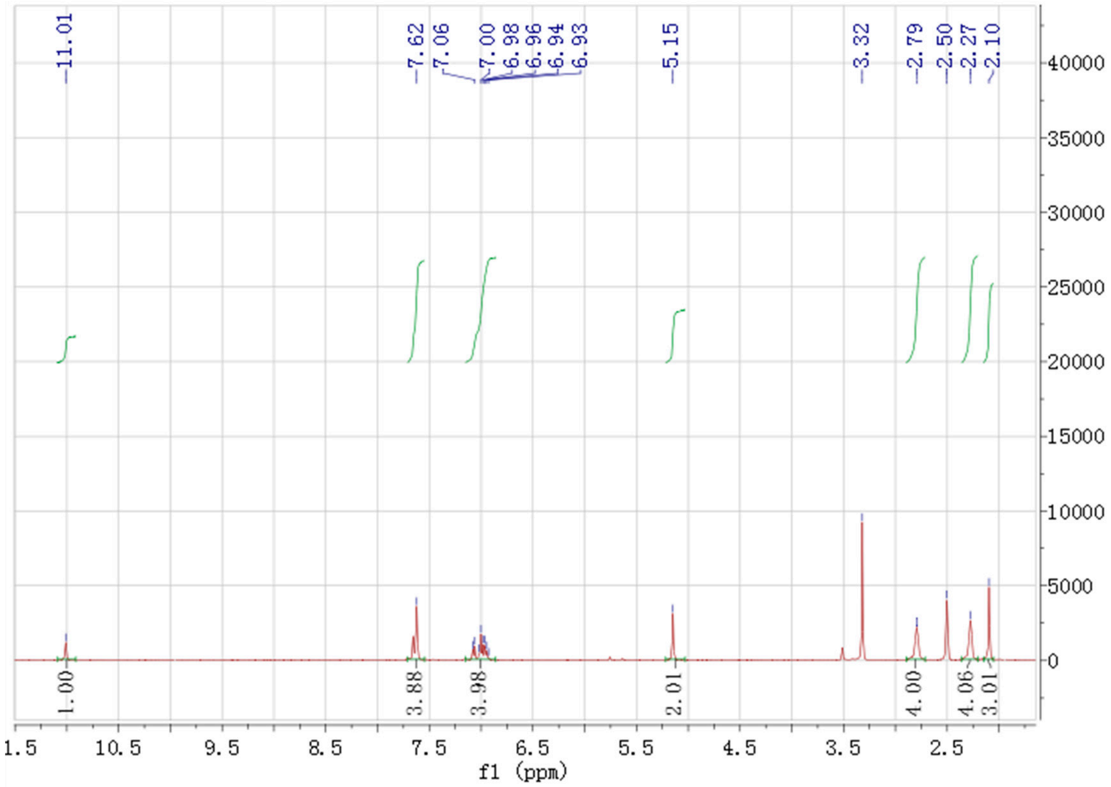

Figure S13. ^{1}H-NMR spectrum of **5b**.

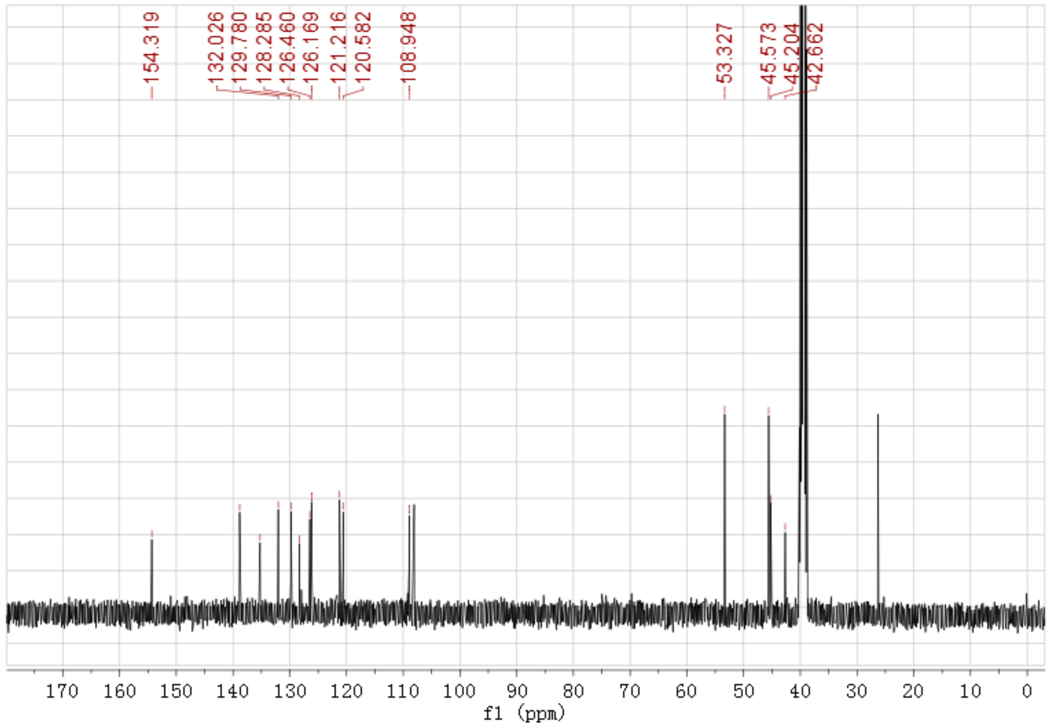

Figure S14. ^{13}C-NMR spectrum of **5b**.

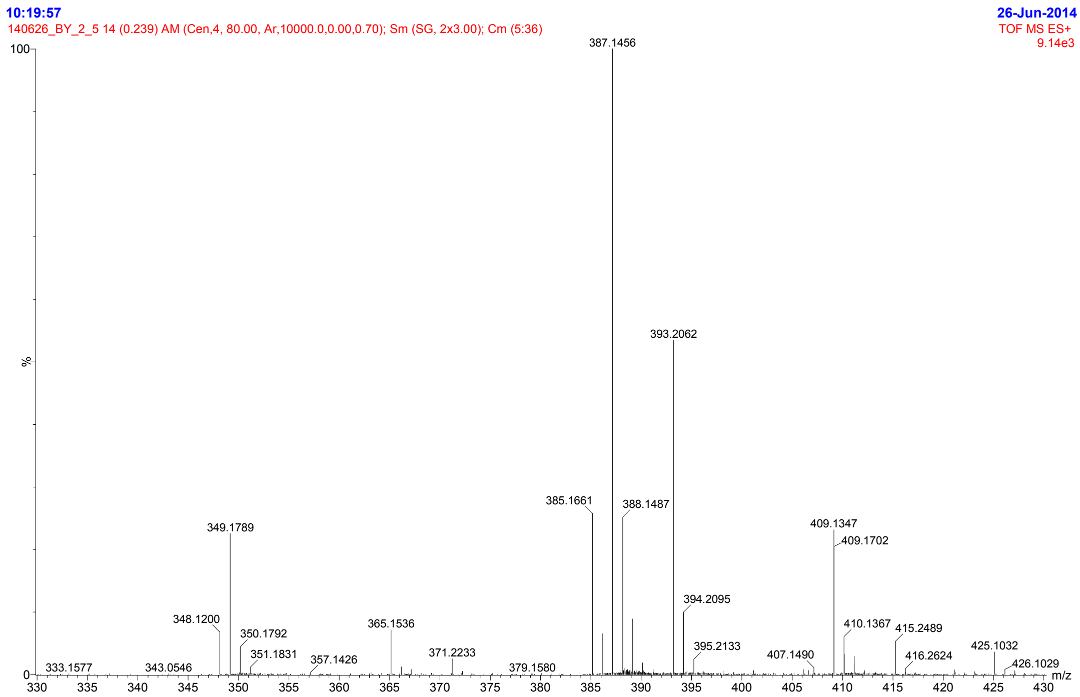

Figure S15. HRMS (EI) spectrum of **5b**.

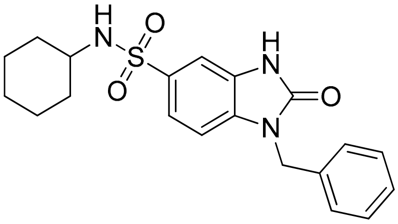

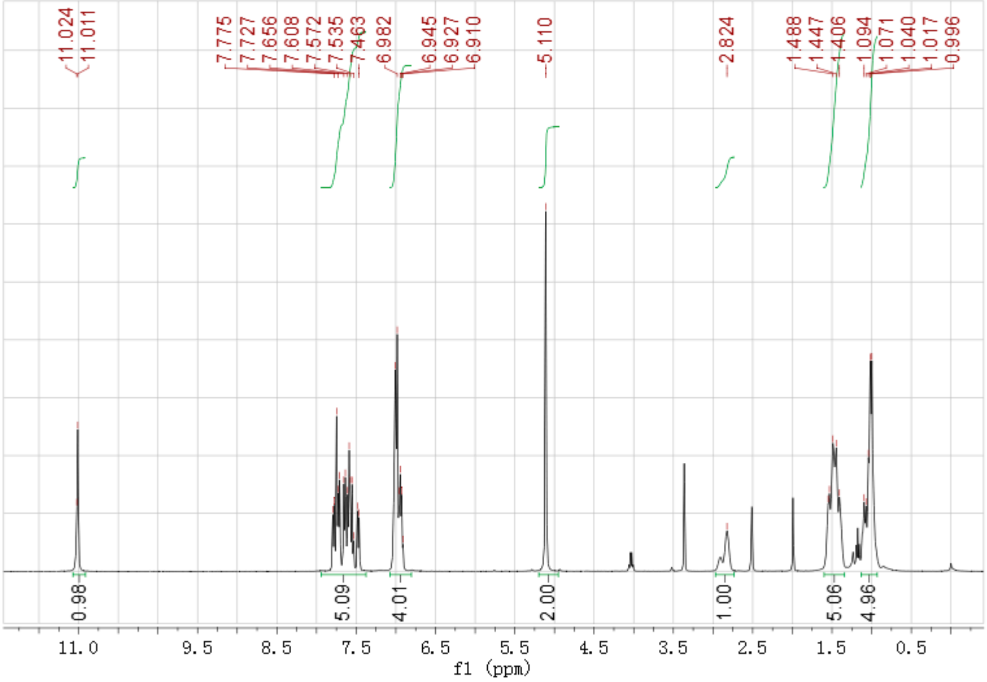

Figure S16. ^{1}H-NMR spectrum of **5c**.

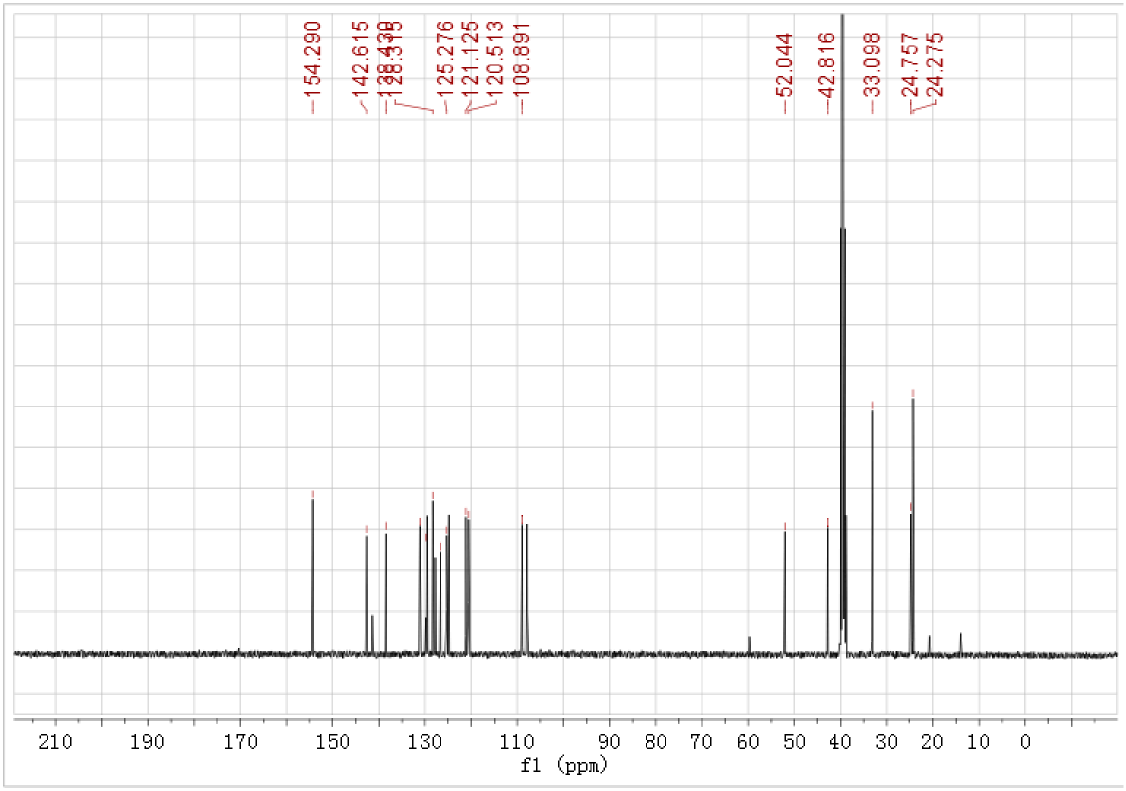

Figure S17. ^{13}C-NMR spectrum of **5c**.

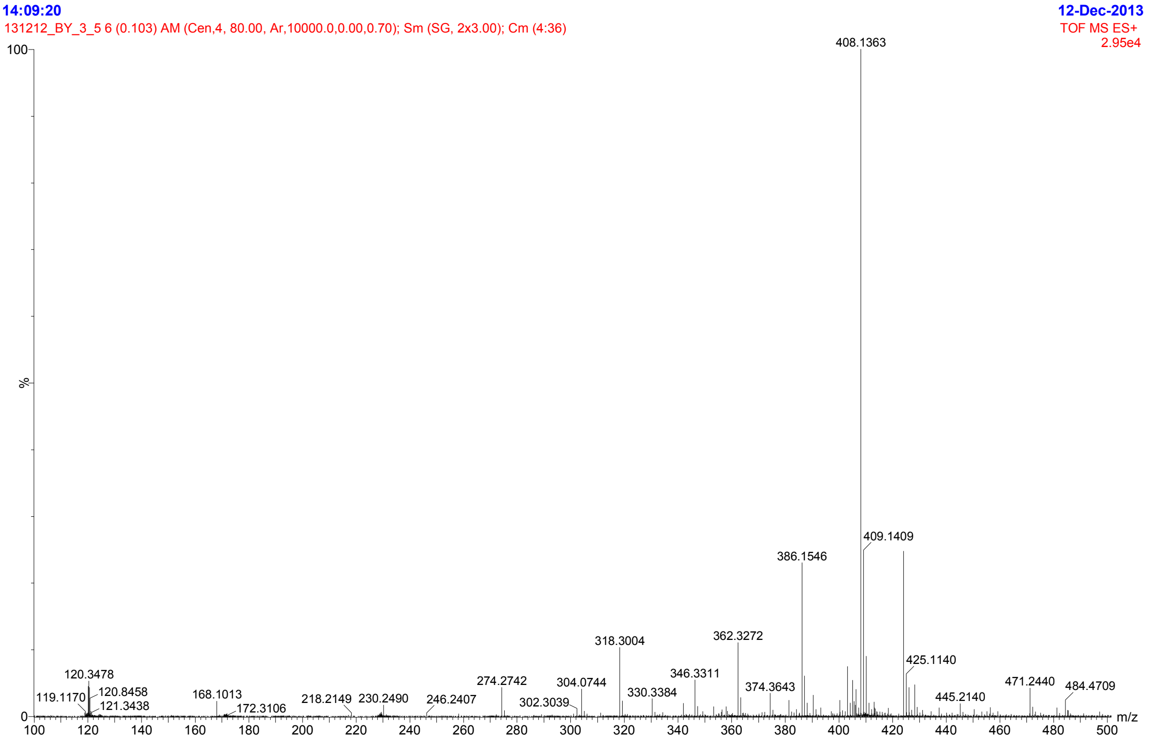

Figure S18. HRMS (EI) spectrum of **5c**.

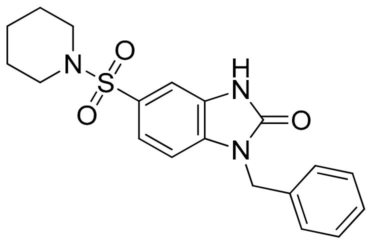

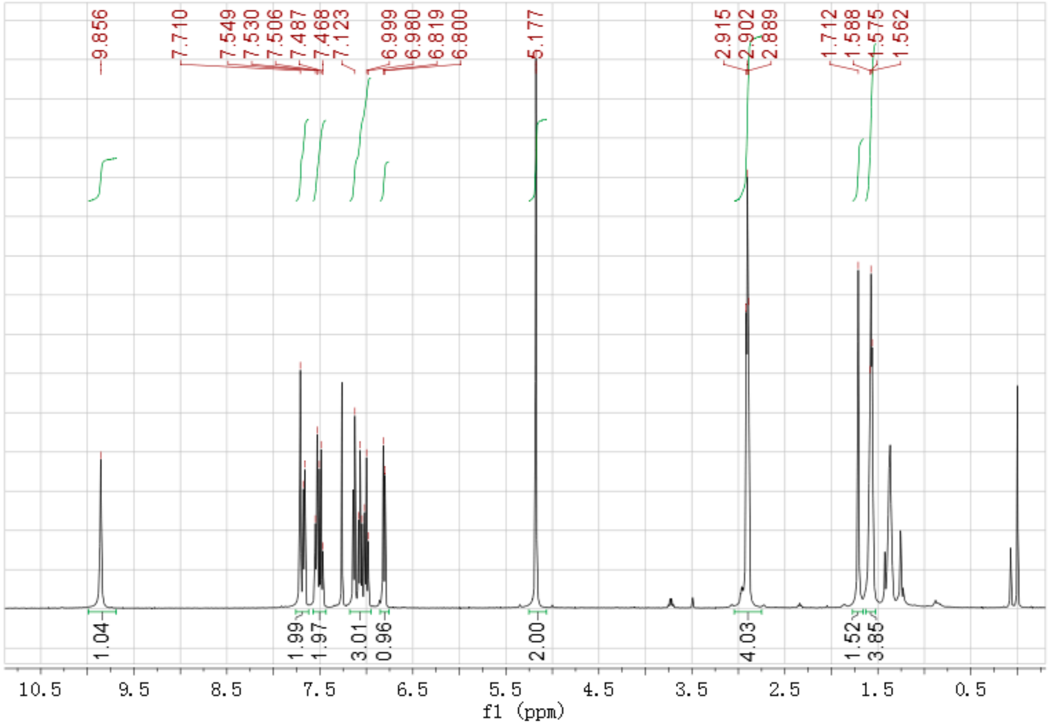

Figure S19. ^{1}H-NMR spectrum of **5d**.

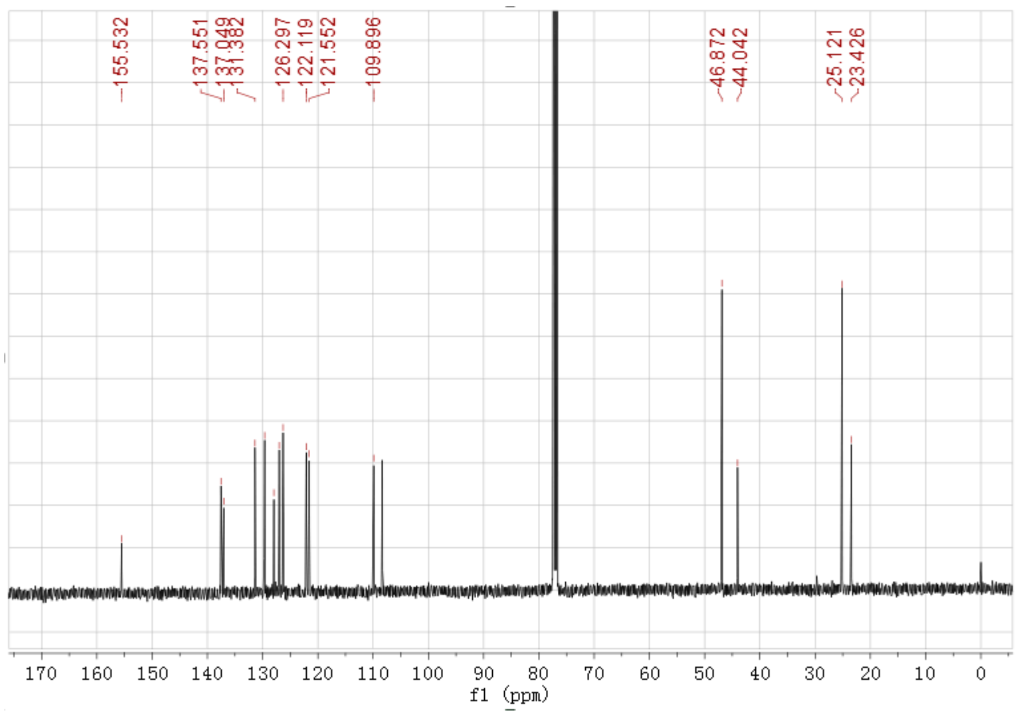

Figure S20. ^{13}C-NMR spectrum of **5d**.

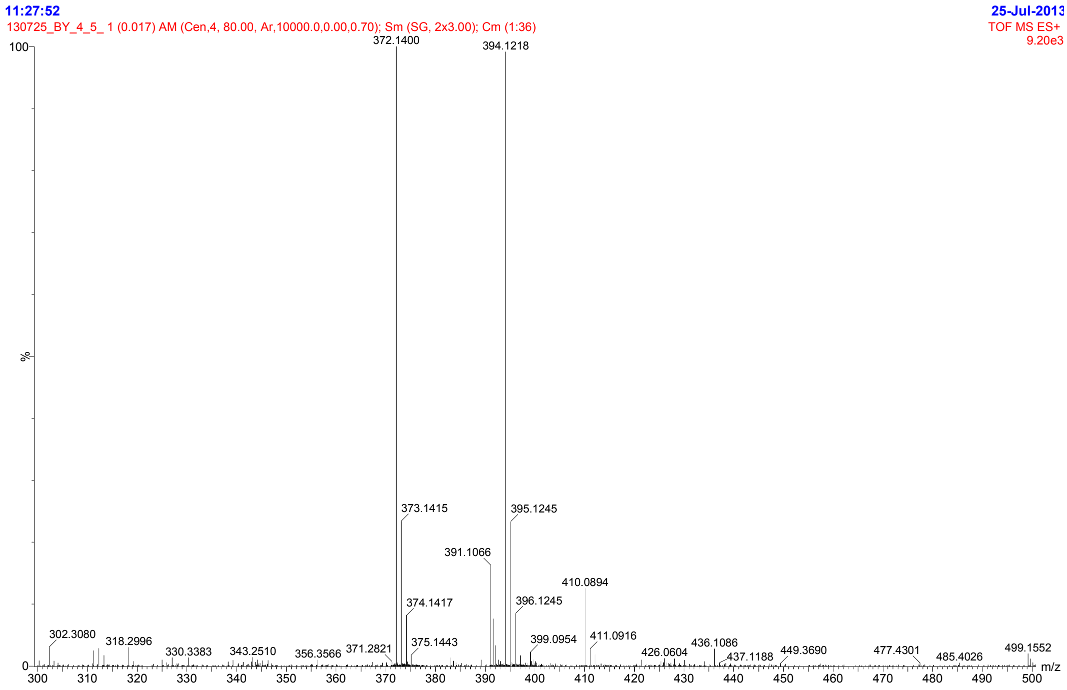

Figure S21. HRMS (EI) spectrum of **5d**.

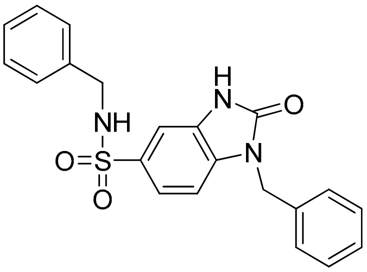

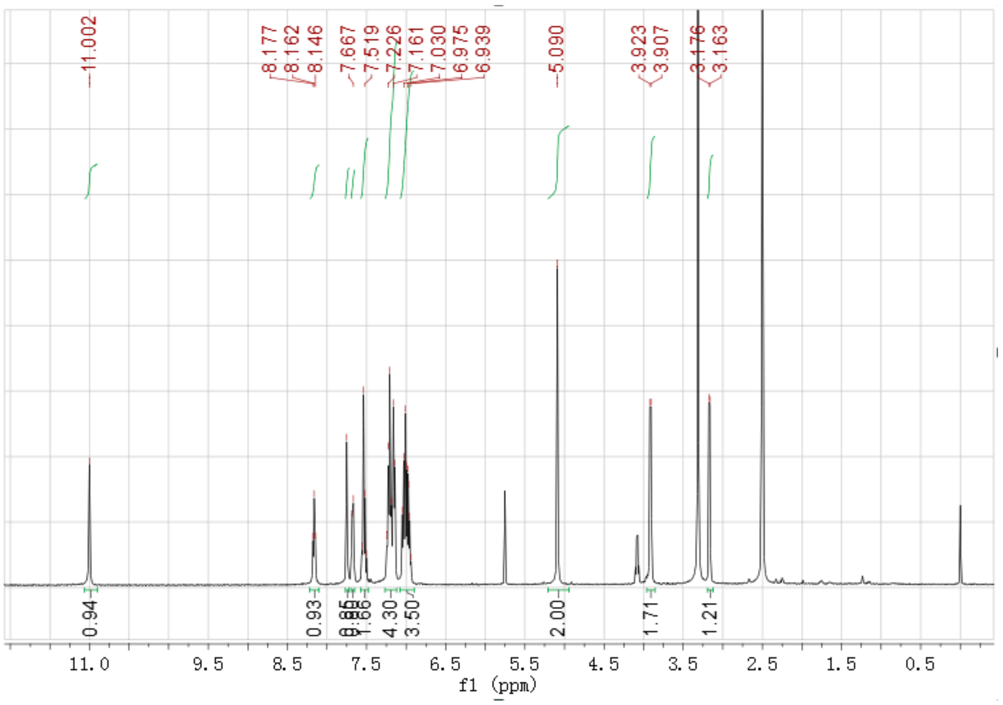

Figure S22. ^{1}H-NMR spectrum of **5e**.

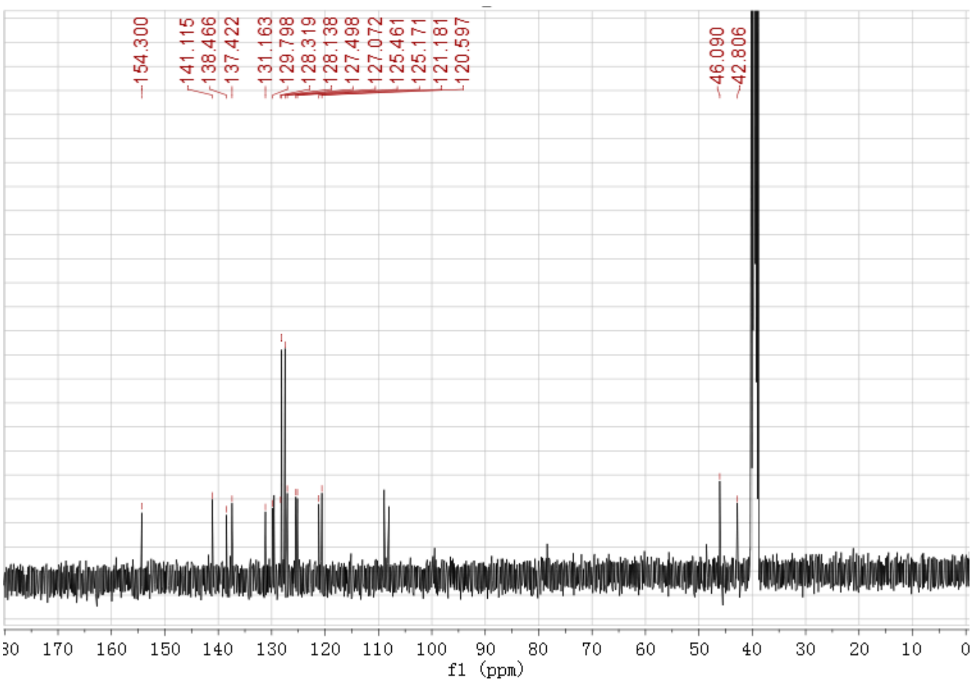

Figure S23. ^{13}C-NMR spectrum of **5e**.

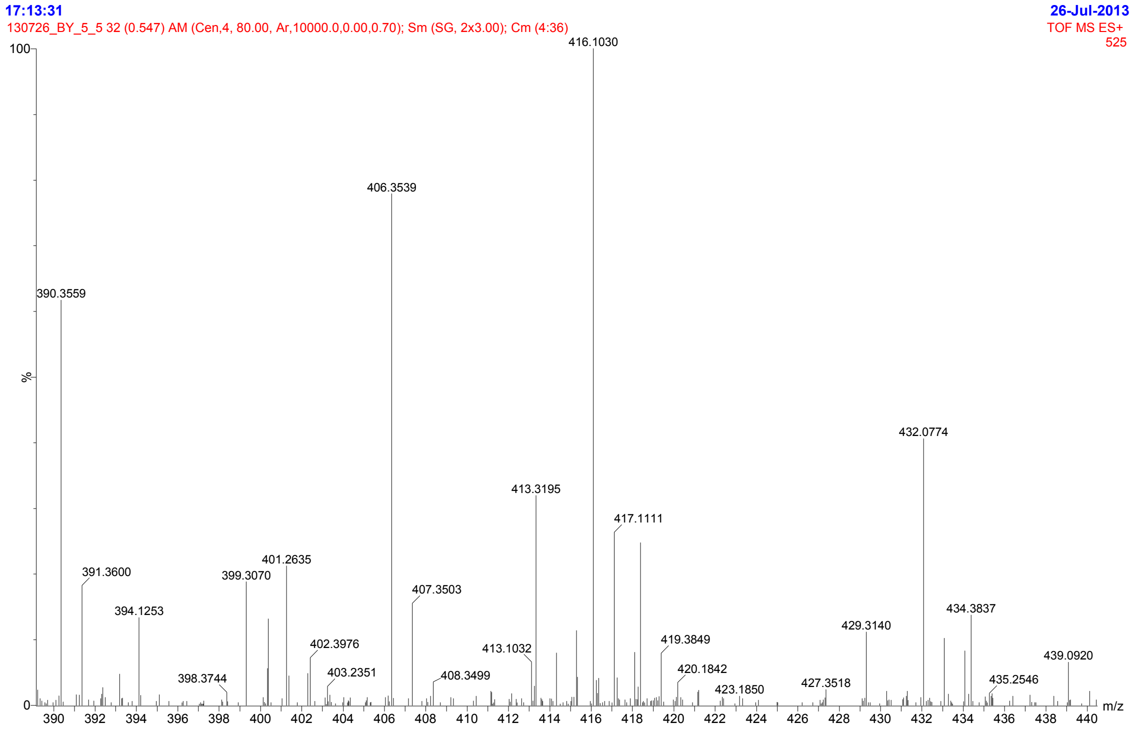

Figure S24. HRMS (EI) spectrum of **5e**.

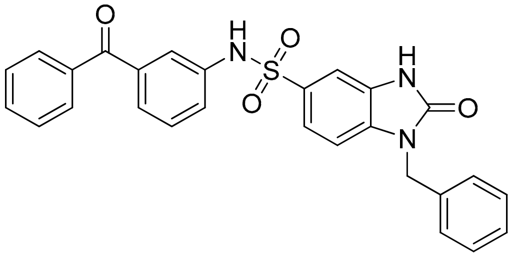

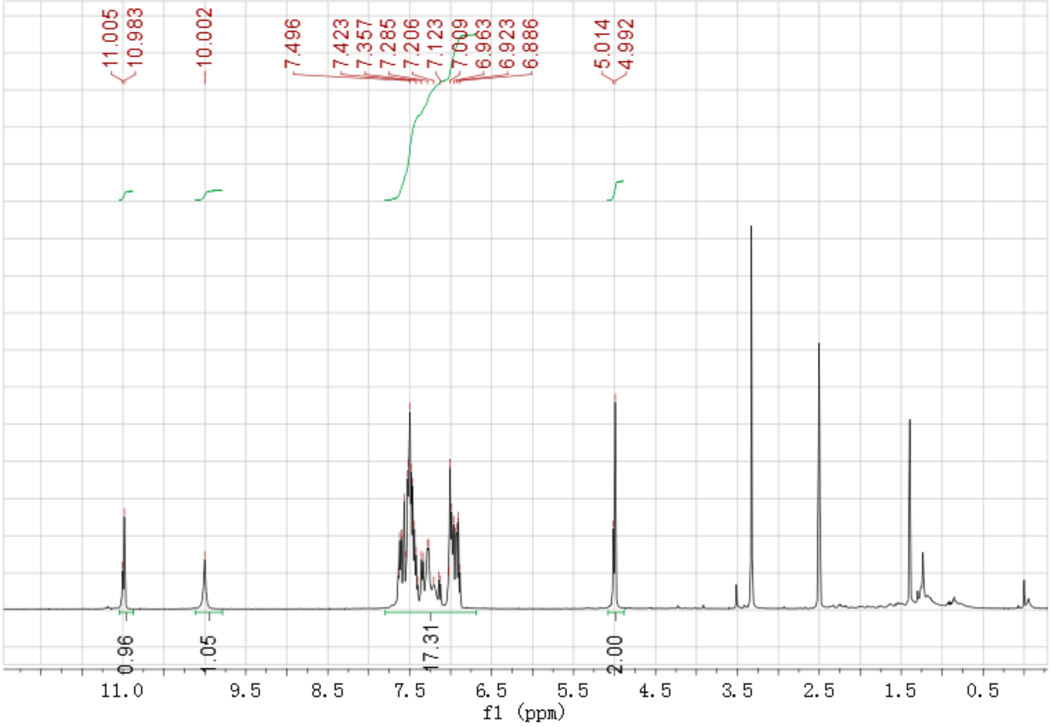

Figure S25. ^{1}H-NMR spectrum of **5f**.

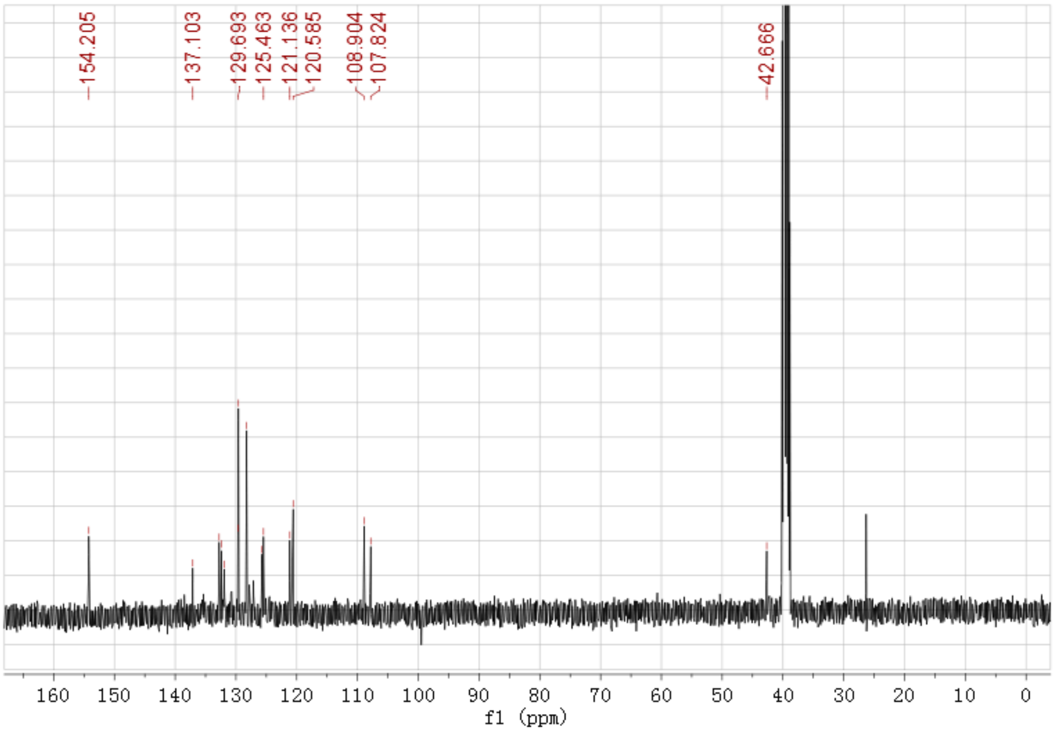

Figure S26. ^{13}C-NMR spectrum of **5f**.

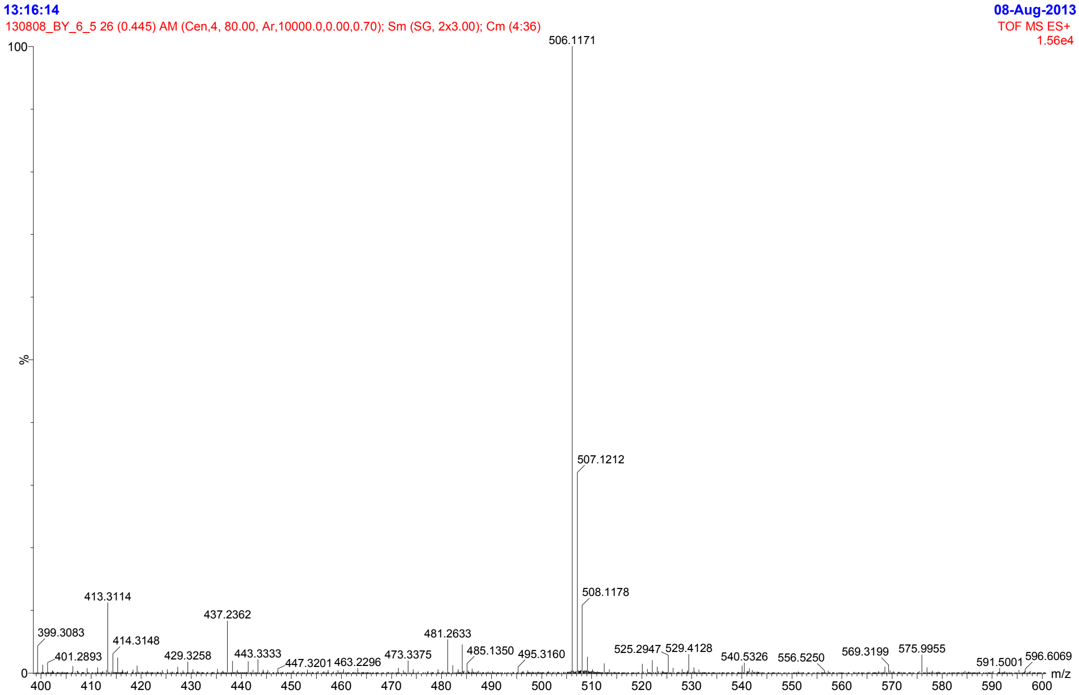

Figure 27. HRMS (EI) spectrum of **5f**.

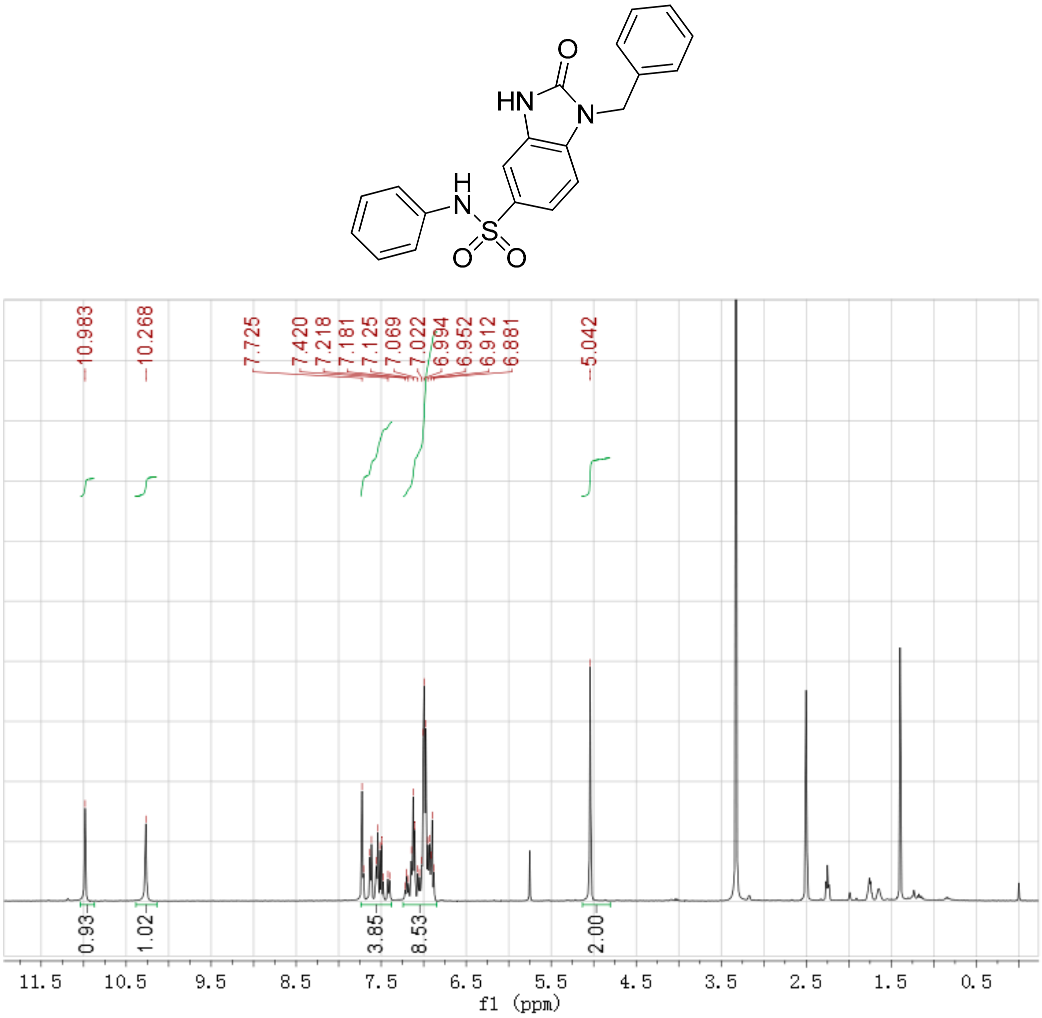

Figure S28. ^{1}H-NMR spectrum of **5g**.

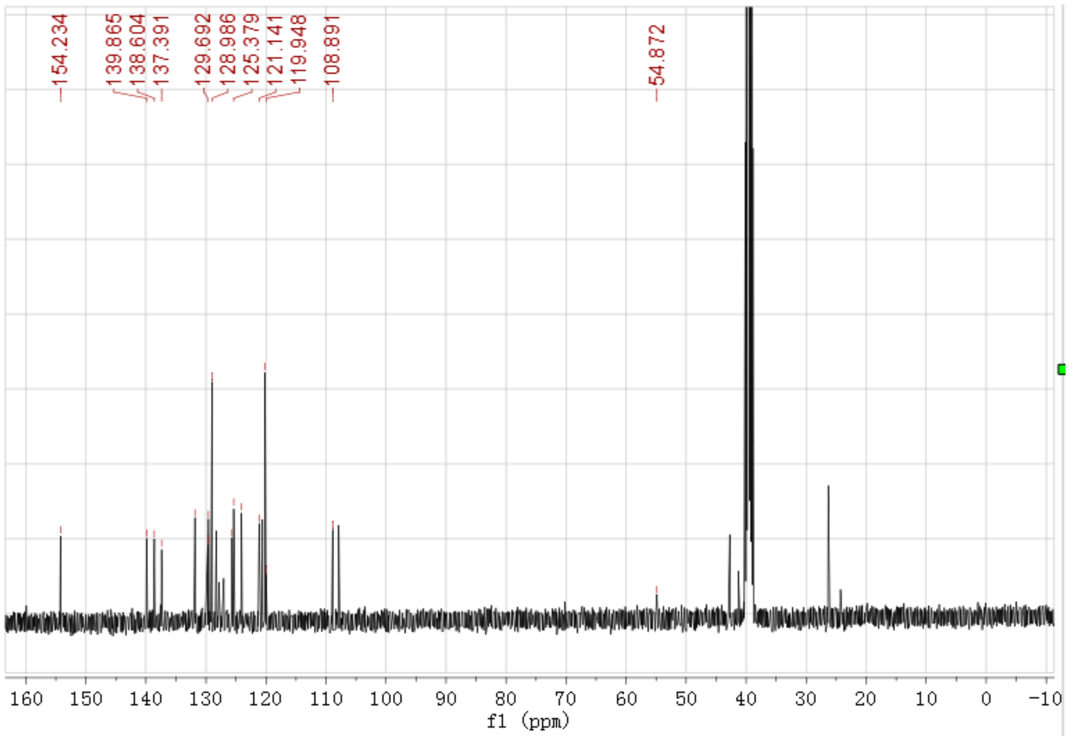

Figure S29. ^{13}C-NMR spectrum of **5g**.

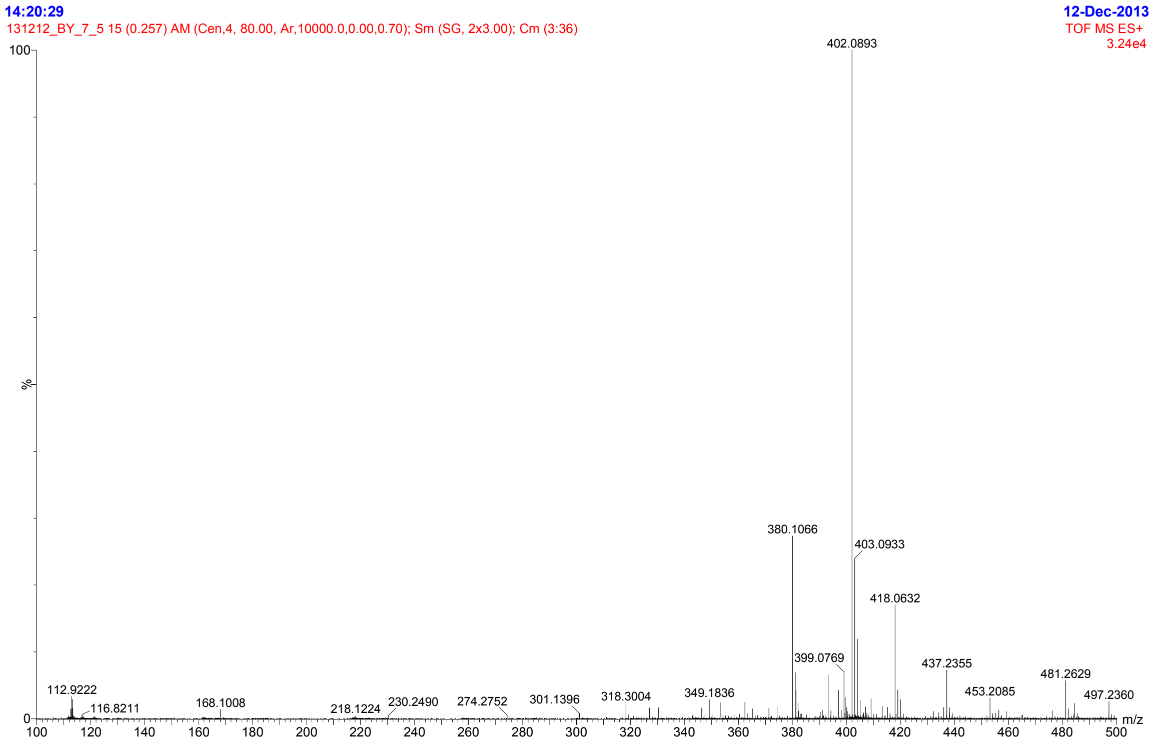

Figure S30. HRMS (EI) spectrum of **5g**.

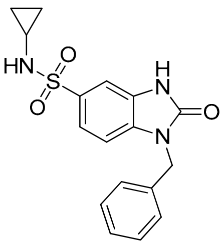

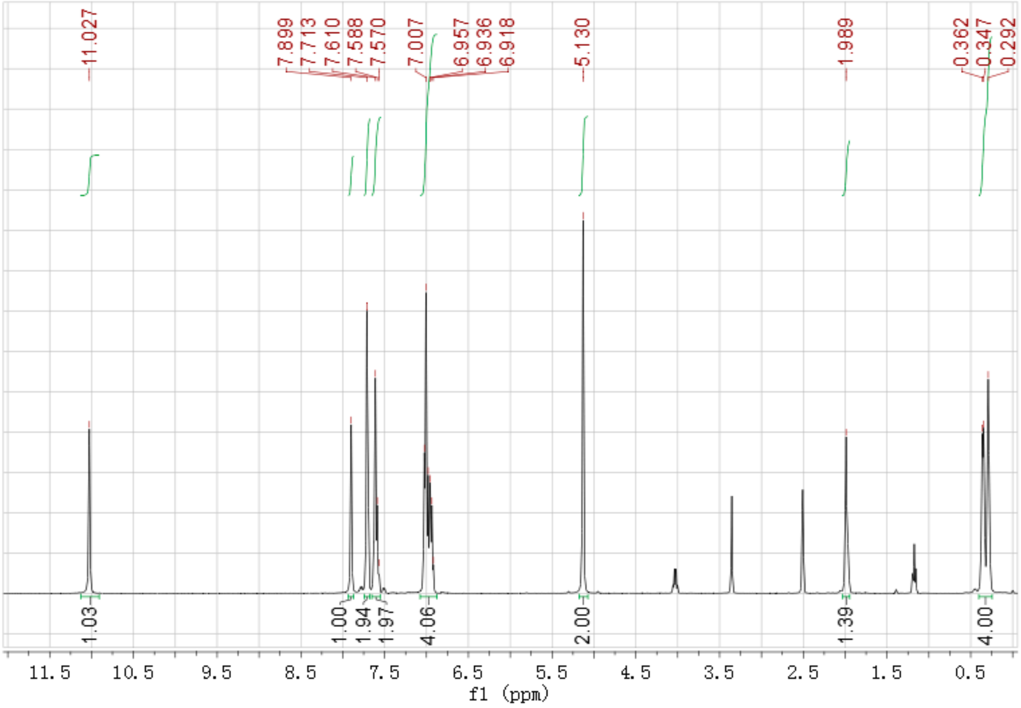

Figure S31. ^{1}H-NMR spectrum of **5h**.

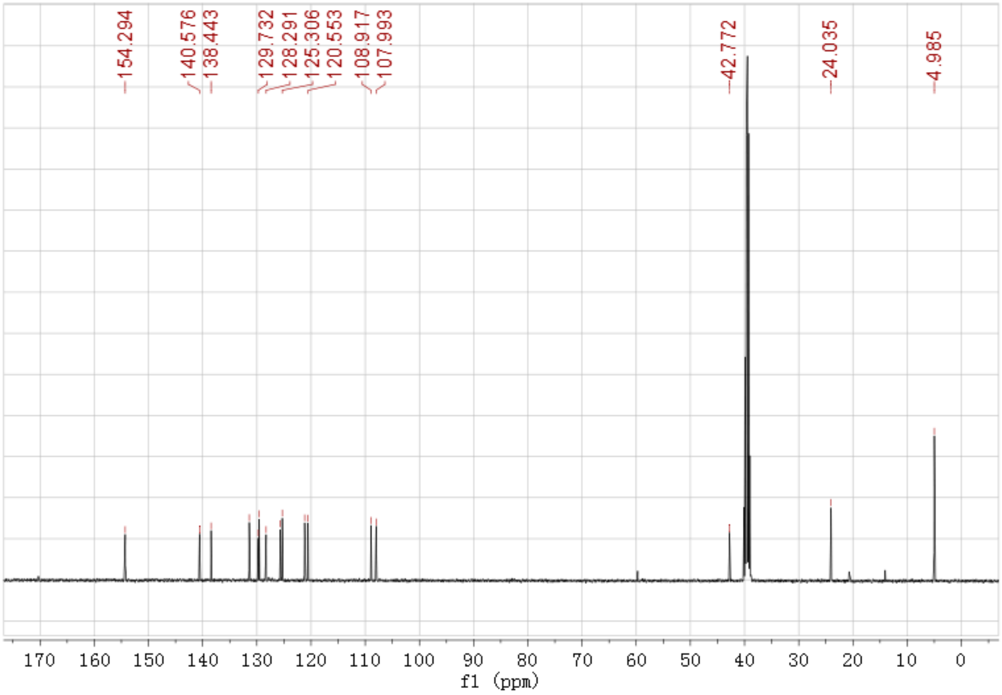

Figure S32. ^{13}C-NMR spectrum of **5h**.

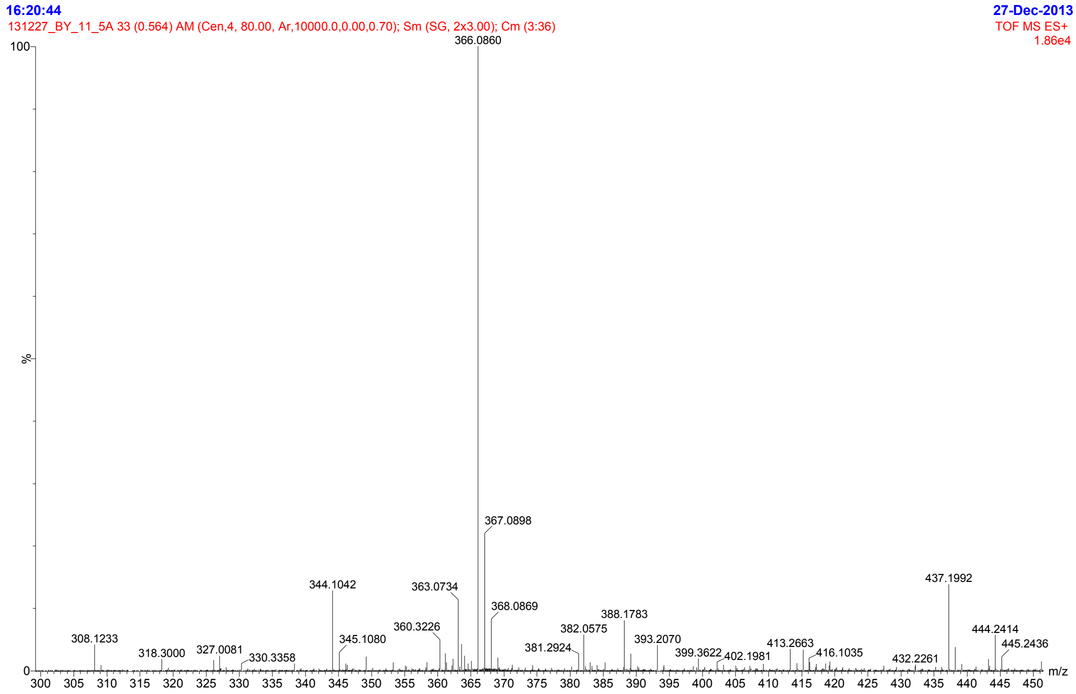

Figure S33. HRMS (EI) spectrum of **5h**.

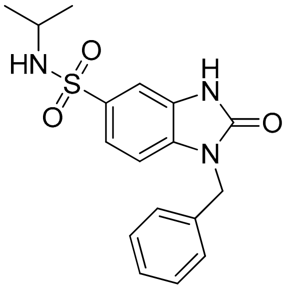

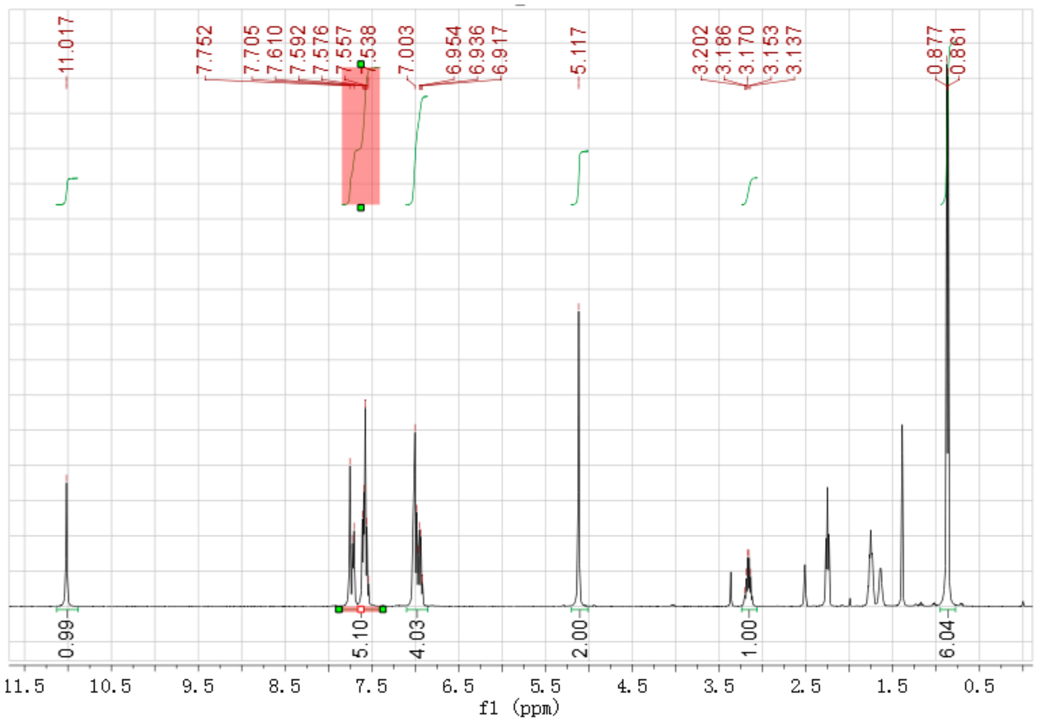

Figure S34. ^{1}H-NMR spectrum of **5i**.

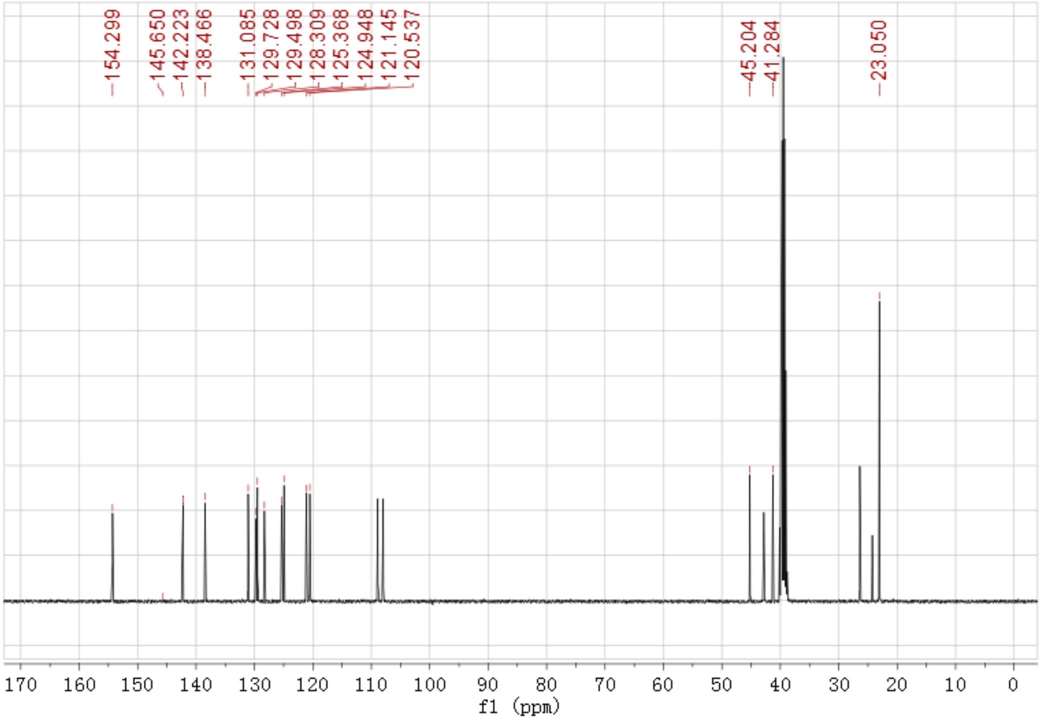

Figure S35. ^{13}C-NMR spectrum of **5i**.

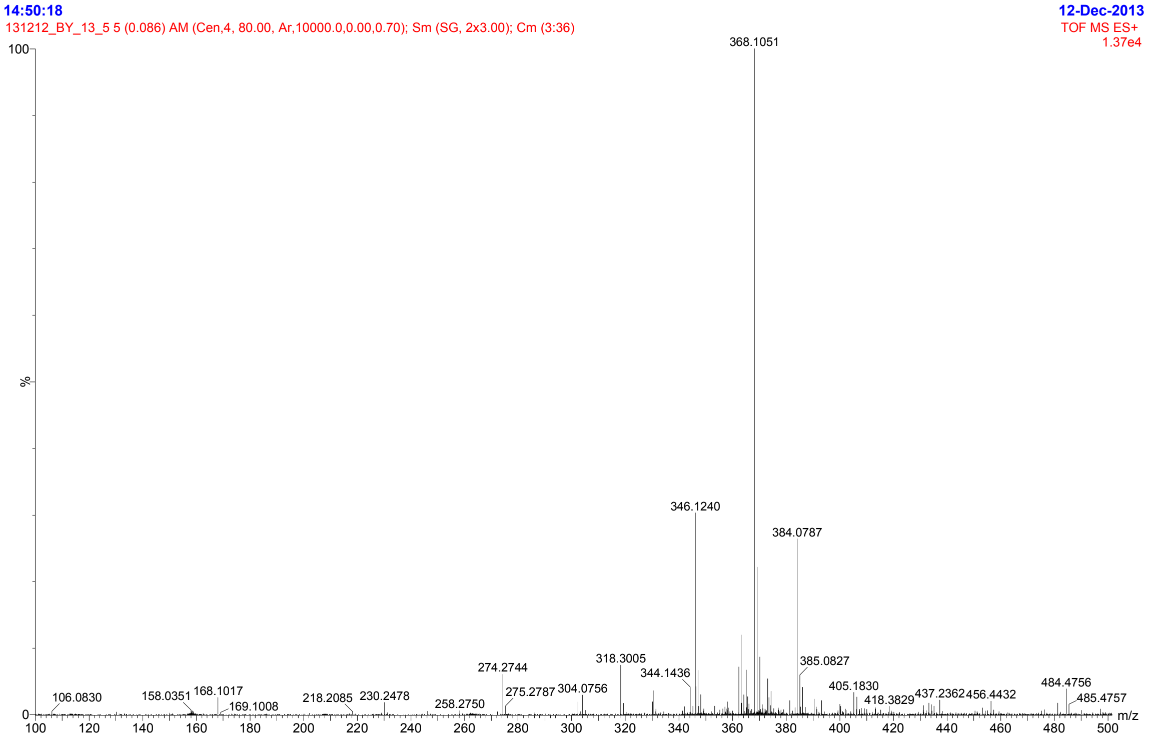

Figure S36. HRMS (EI) spectrum of **5i**.

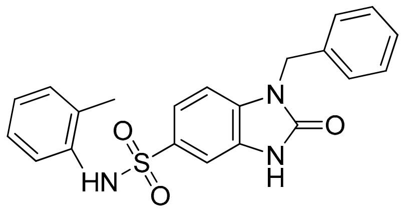

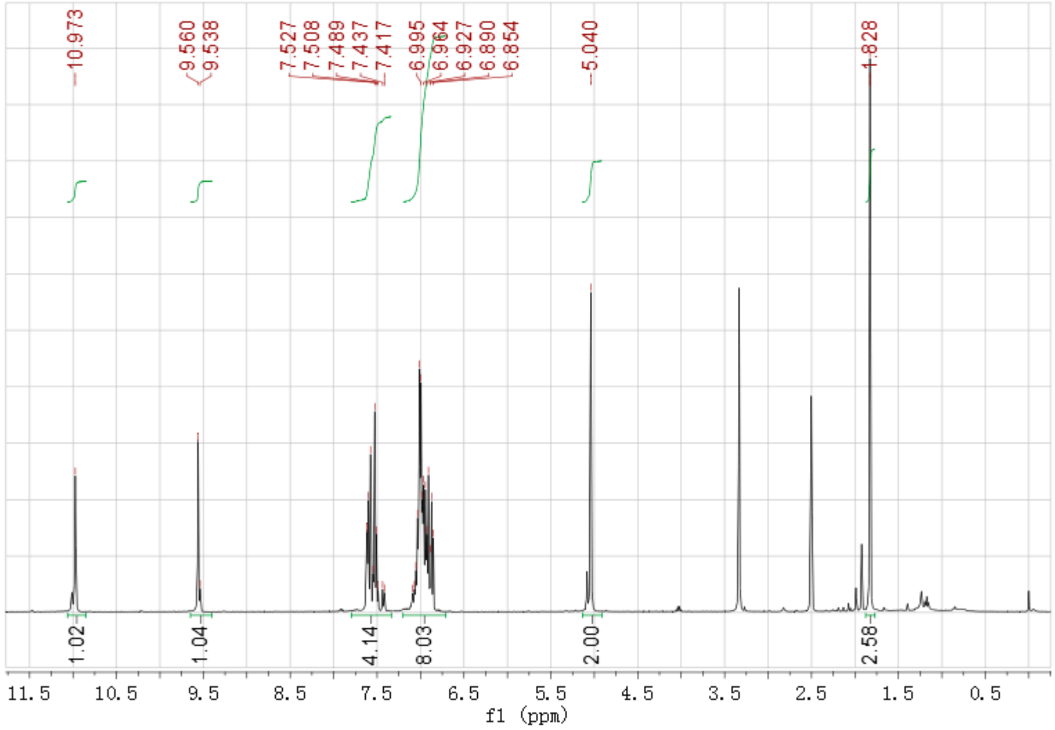

Figure S37. ^{1}H-NMR spectrum of **5j**.

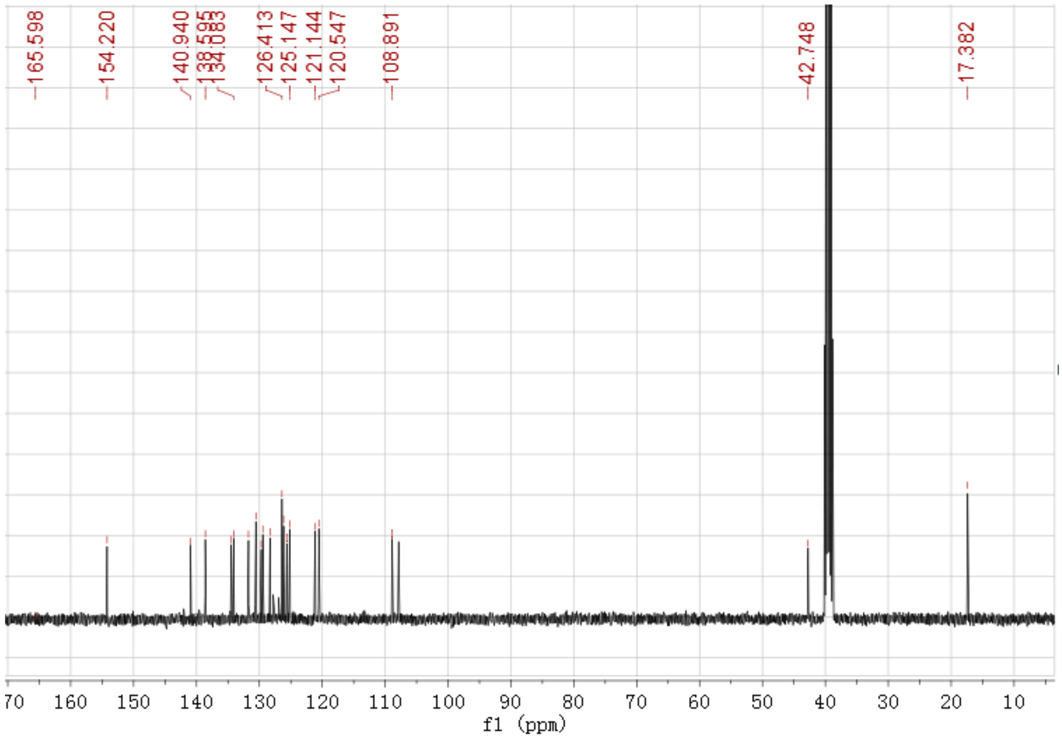

Figure S38. ^{13}C-NMR spectrum of **5j**.

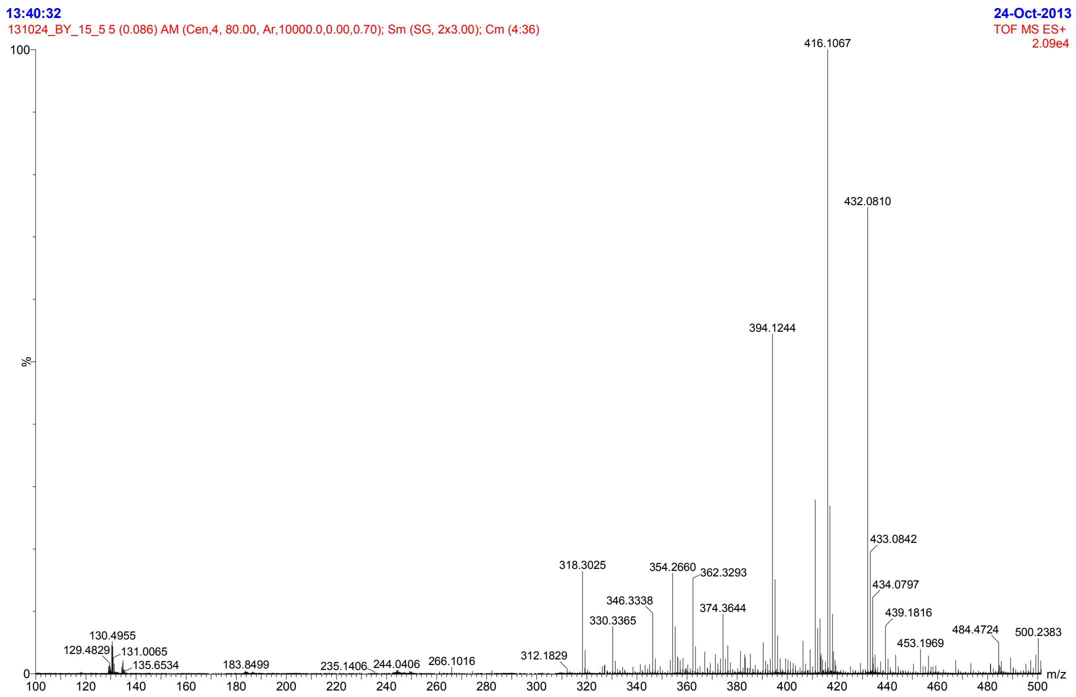

Figure S39. HRMS (EI) spectrum of **5j**.

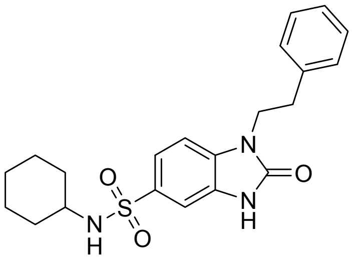

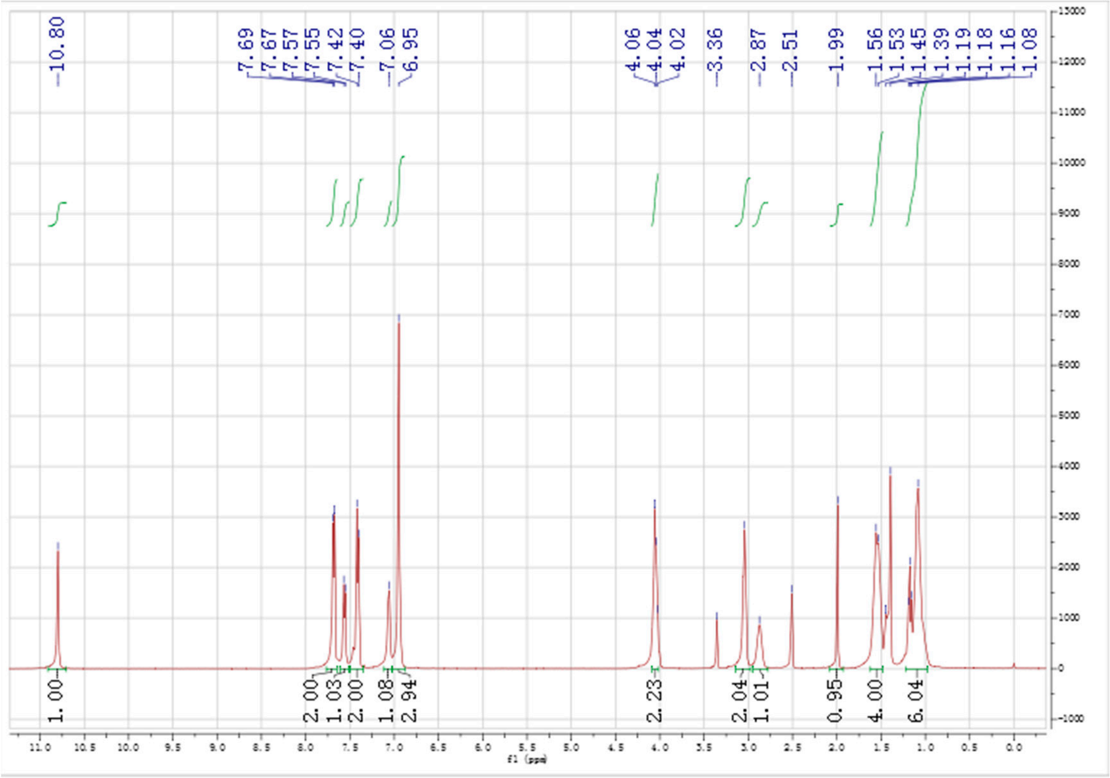

Figure S40. ^{1}H-NMR spectrum of **5k**.

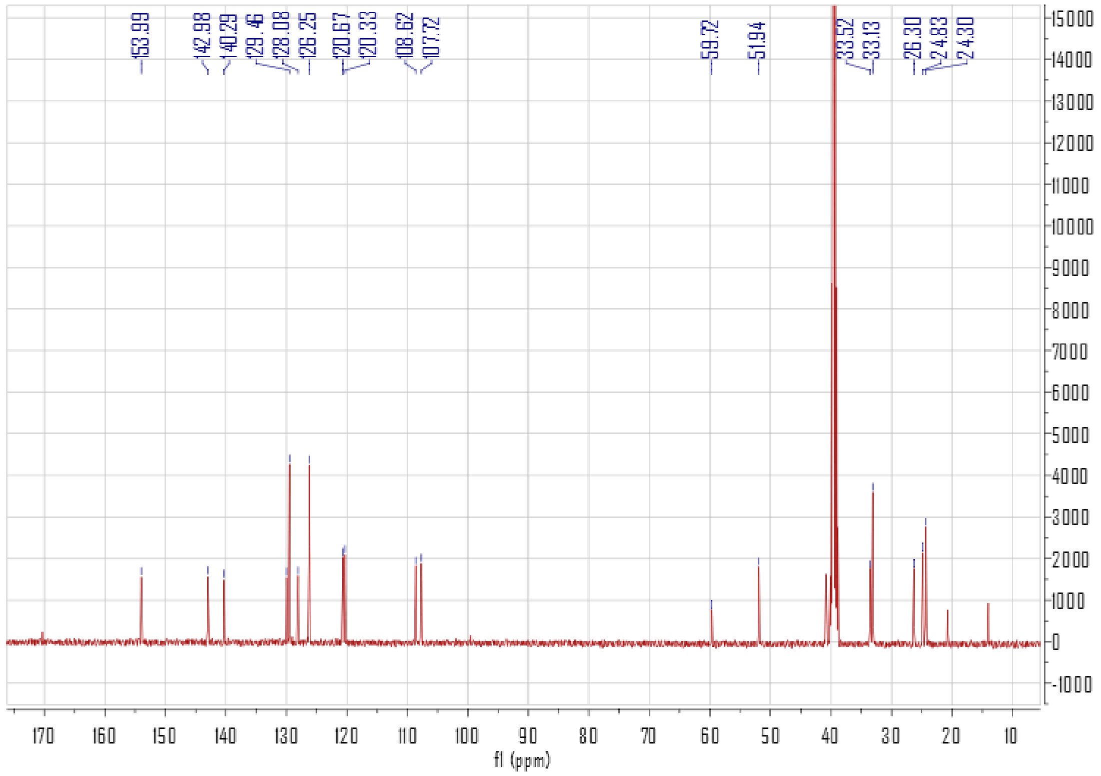

Figure S41. ^{13}C-NMR spectrum of **5k**.

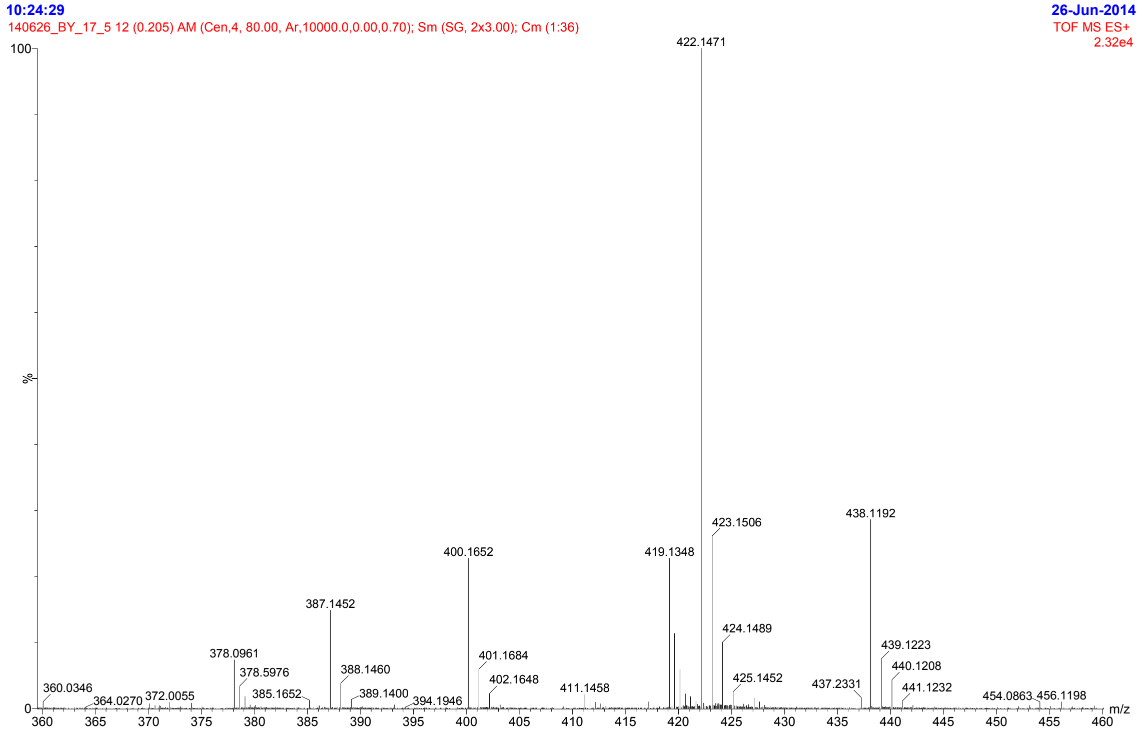

Figure S42. HRMS (EI) spectrum of **5k**.

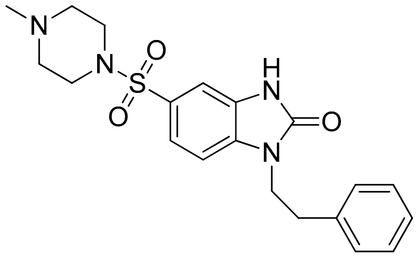

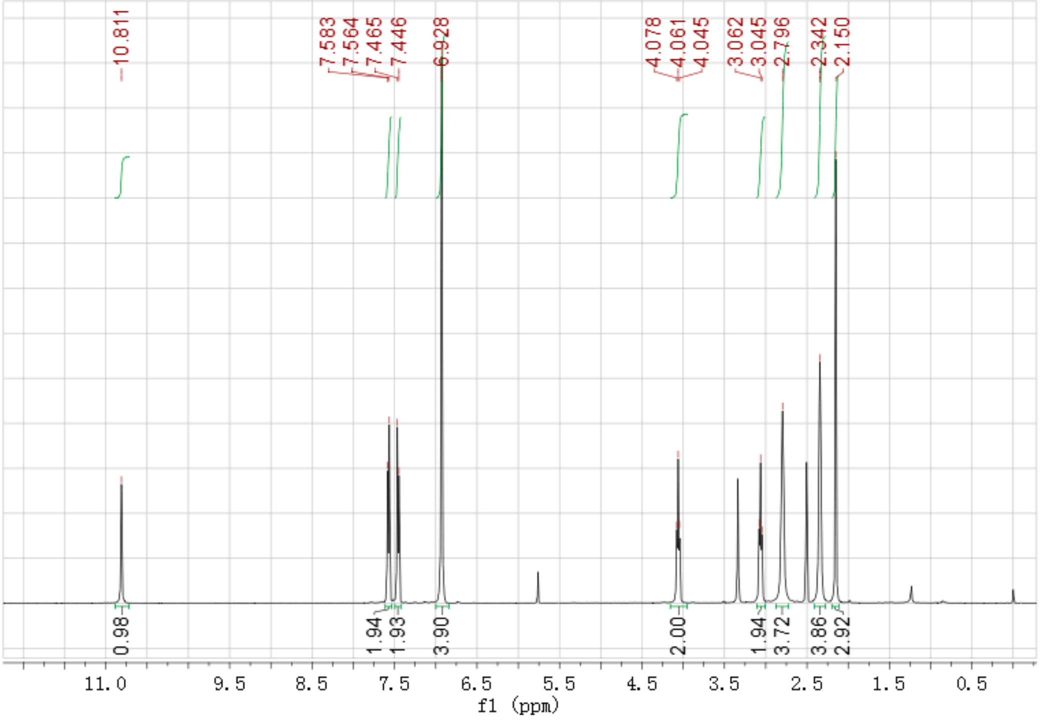

Figure S43. ^{1}H-NMR spectrum of **5l**.

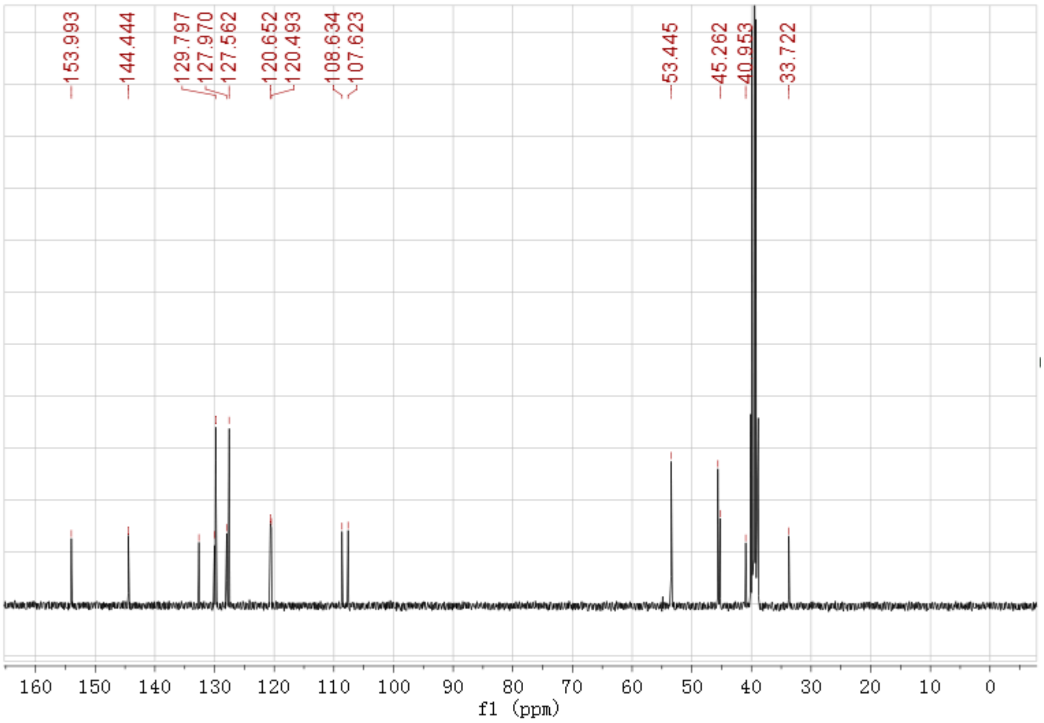

Figure S44. ^{13}C-NMR spectrum of **5l**.

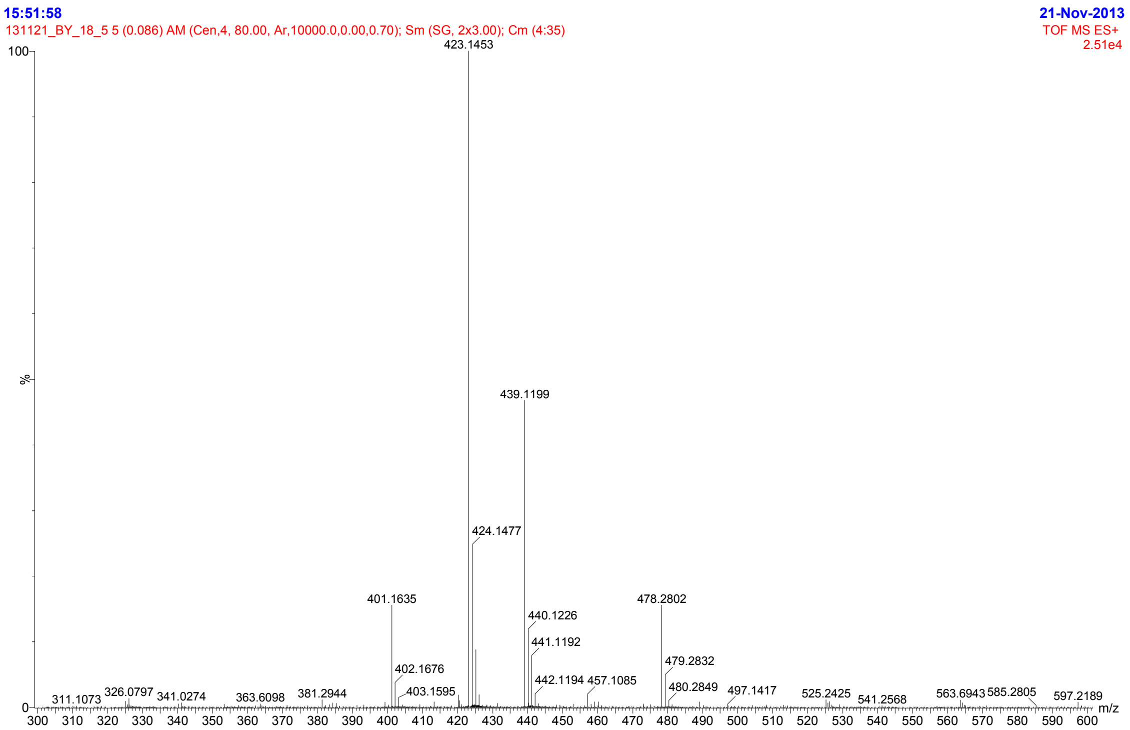

Figure S45. HRMS (EI) spectrum of **5l**.

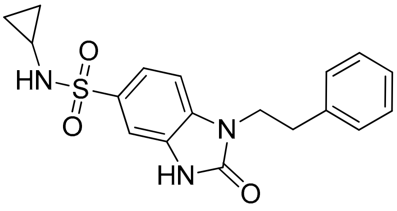

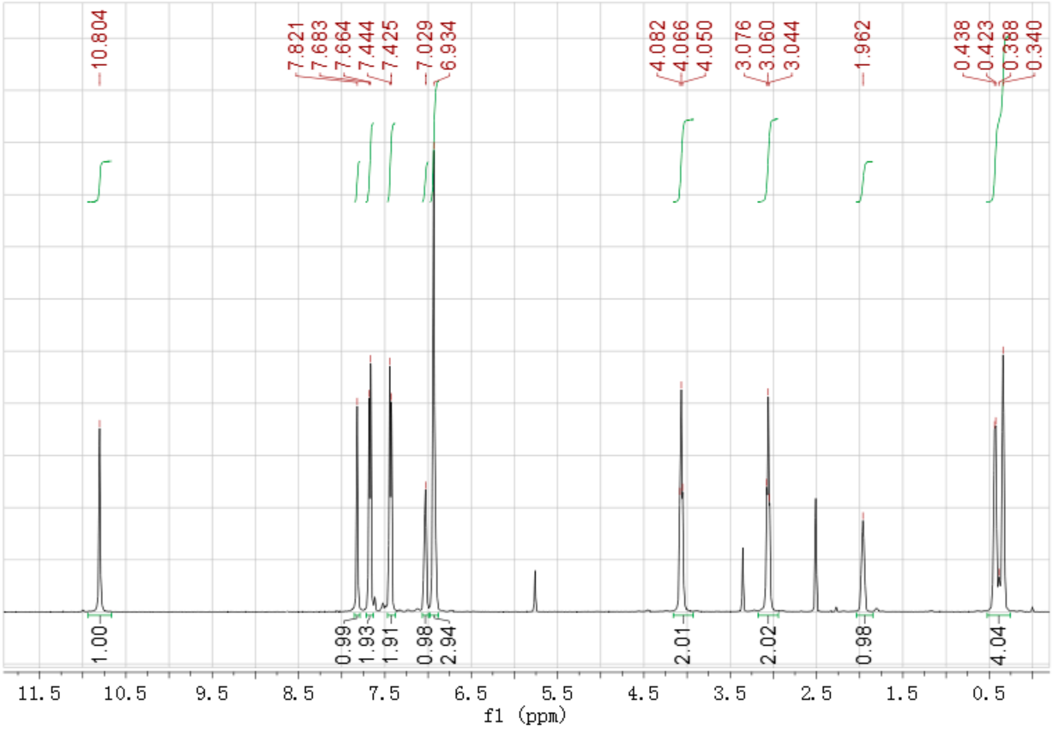

Figure S46. ^{1}H-NMR spectrum of **5m**.

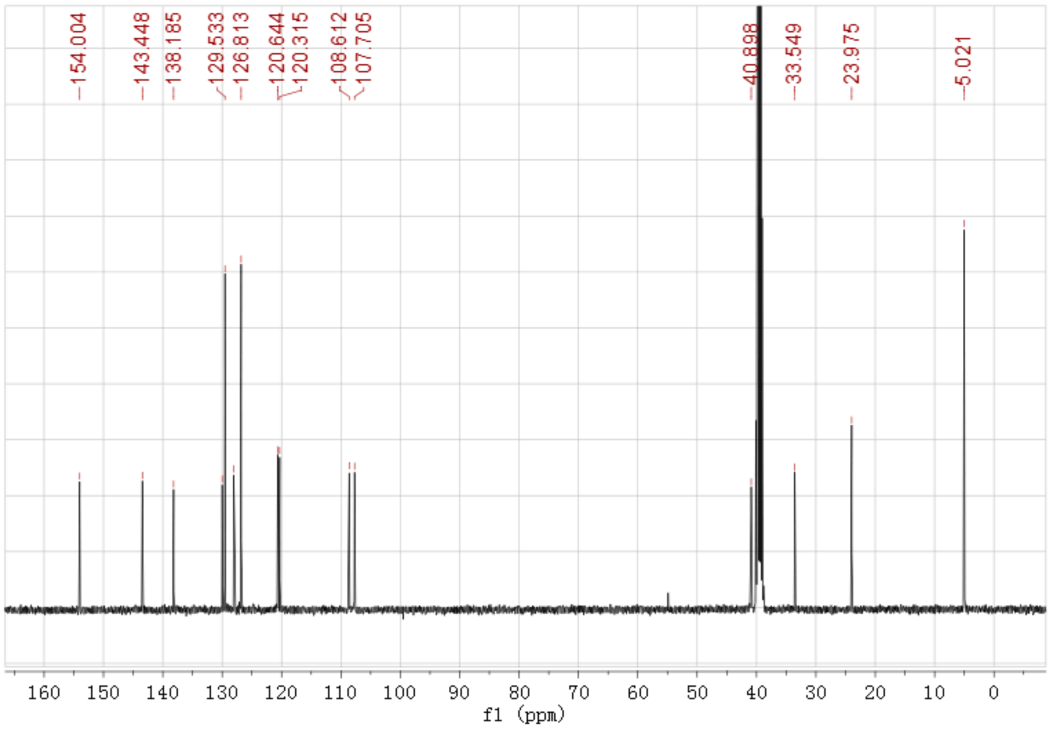

Figure S47. ^{13}C-NMR spectrum of **5m**.

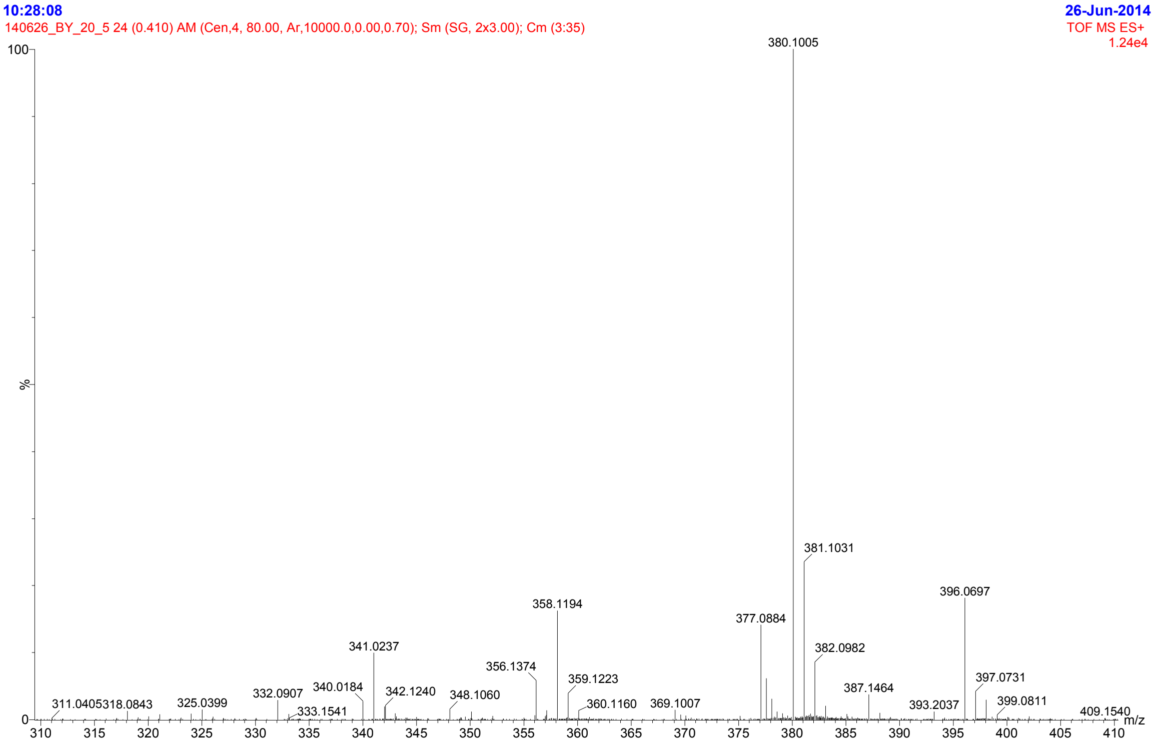

Figure S48. HRMS (EI) spectrum of **5m**.

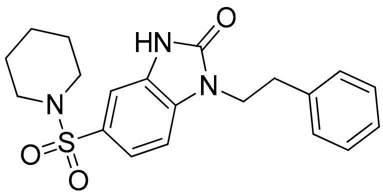

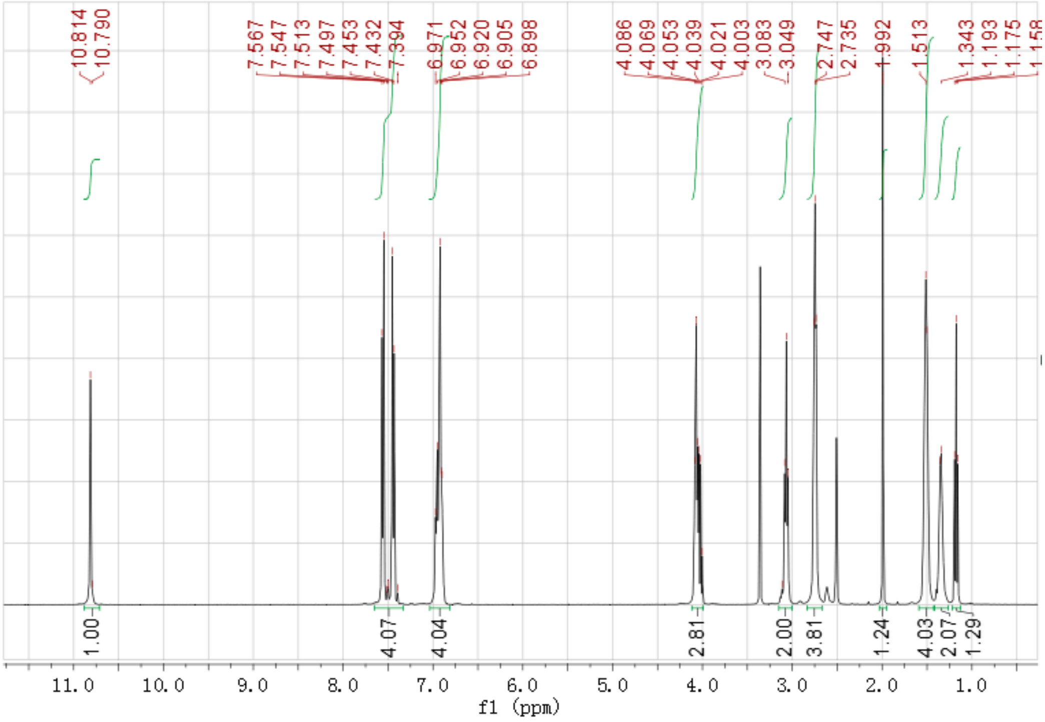

Figure S49. ^{1}H-NMR spectrum of **5n**.

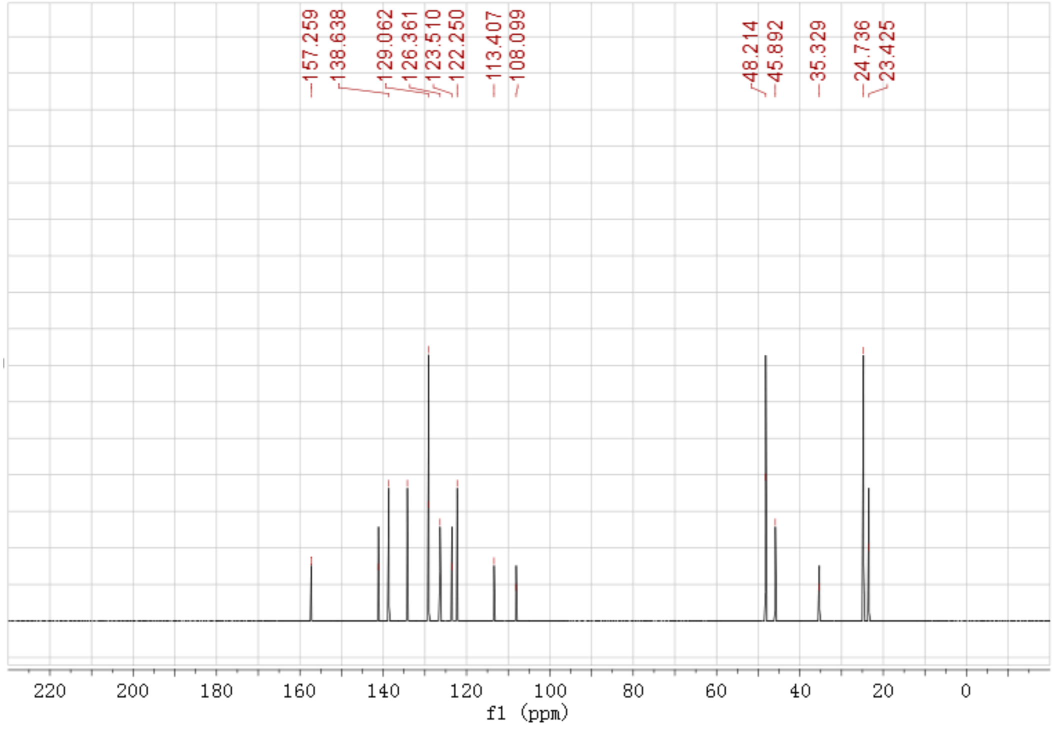

Figure S50. ^{13}C-NMR spectrum of **5n**.

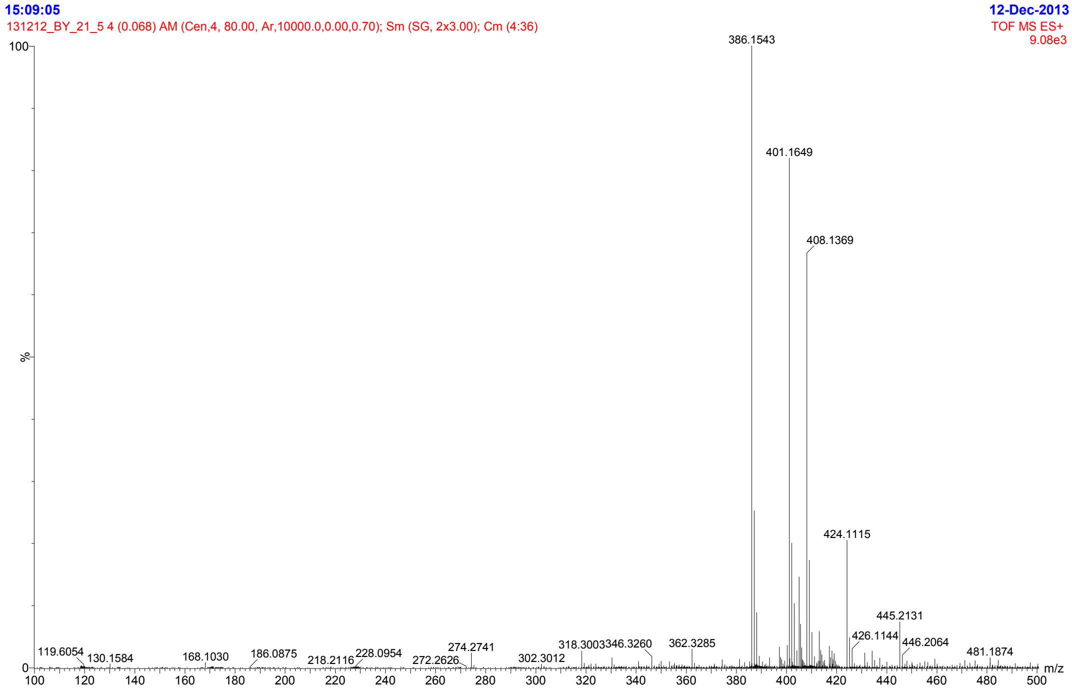

Figure S51. HRMS (EI) spectrum of **5n**.

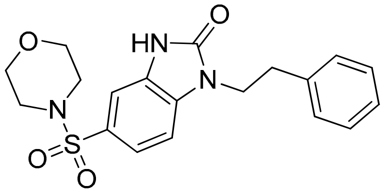

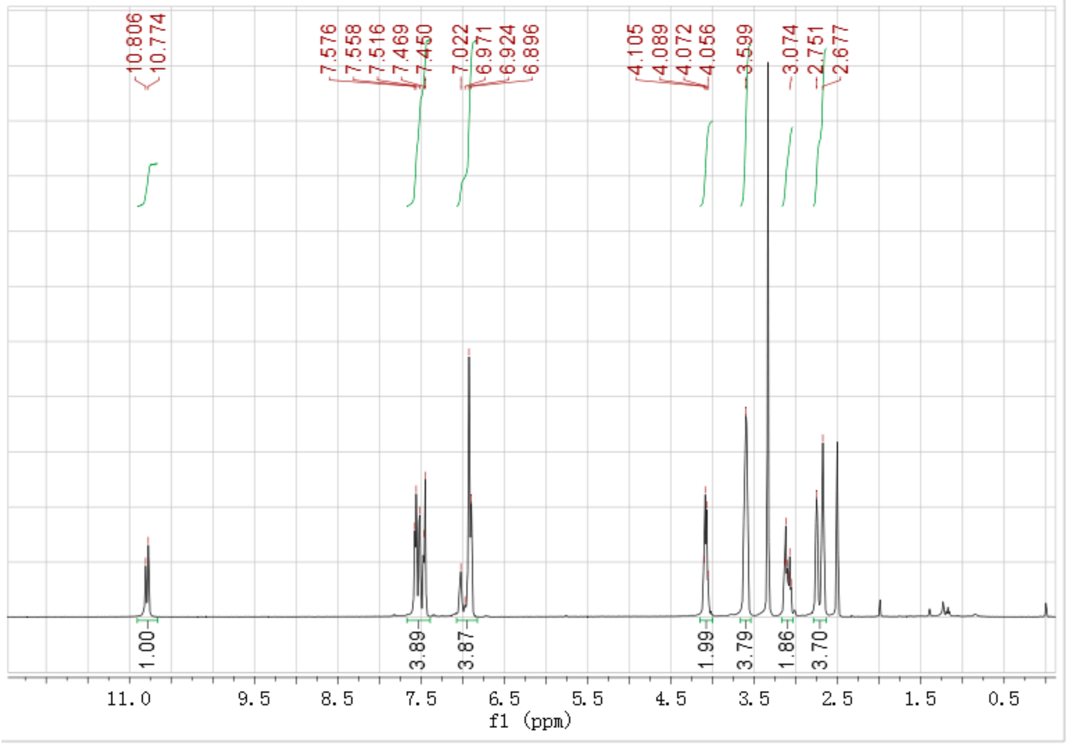

Figure S52. ^{1}H-NMR spectrum of **5o**.

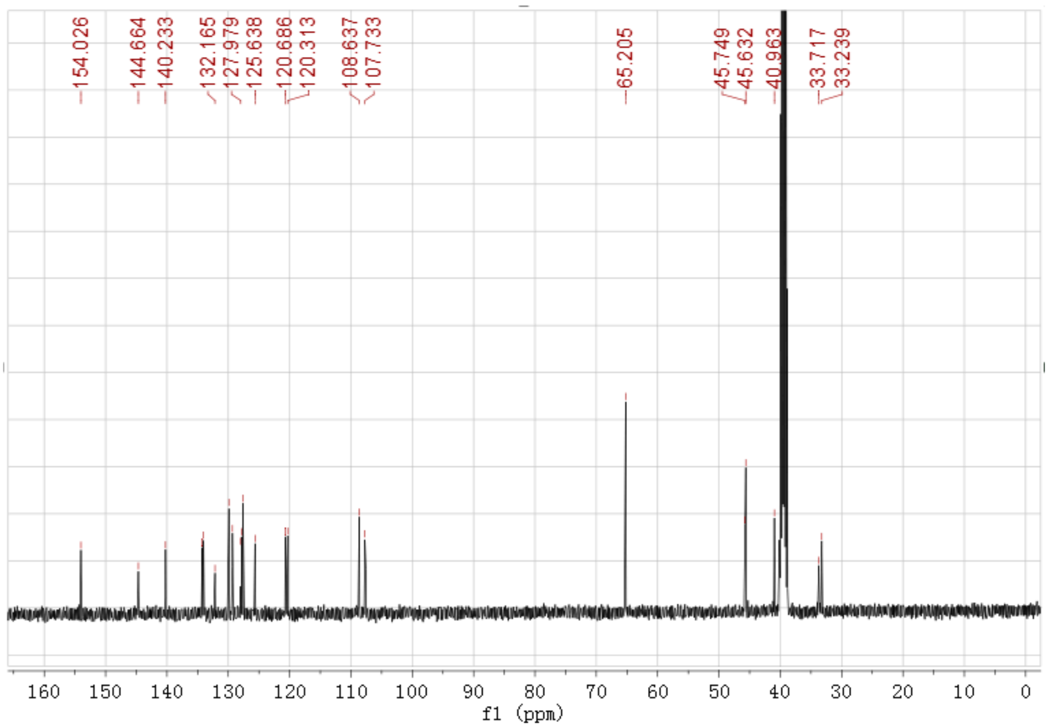

Figure S53. ^{13}C-NMR spectrum of **5o**.

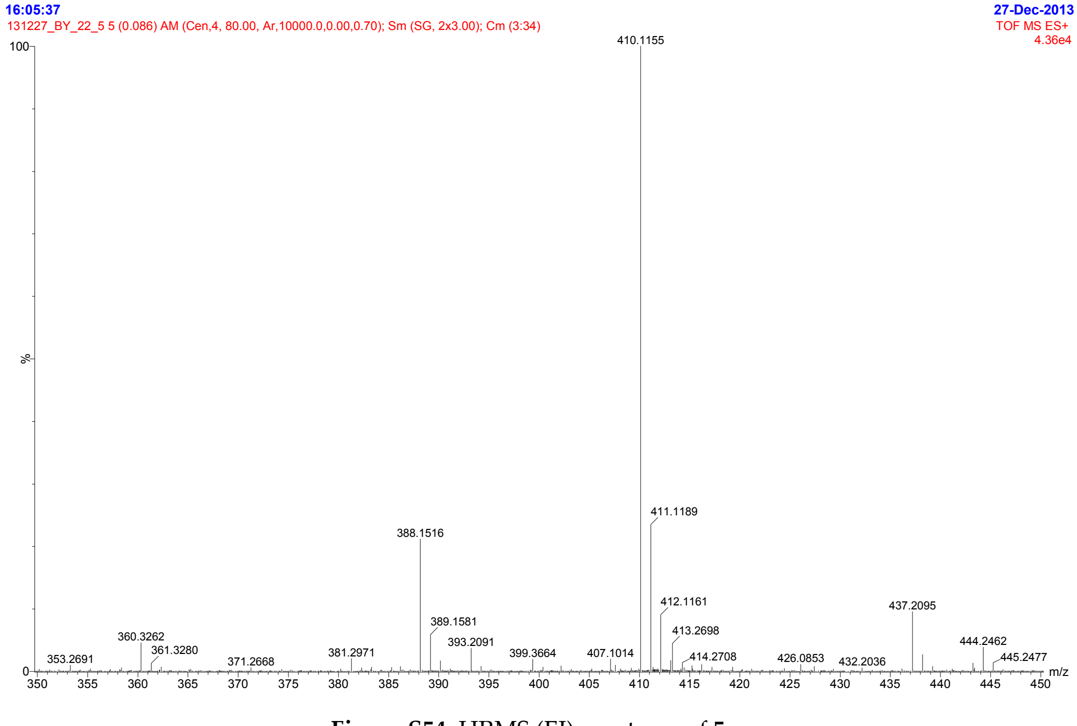

Figure S54. HRMS (EI) spectrum of **5o**.
